# Prevalence of abdominal obesity and associated risk factors among women civil servants in Addis Ababa, Ethiopia, 2021: an institution-based study

**DOI:** 10.1186/s40795-022-00613-9

**Published:** 2022-10-24

**Authors:** Solomon Gebretsadik Bereka, Ayele Worku Demisse, Genanew Kassie Getahun

**Affiliations:** 1 Department of Nutrition Menelik II Medical and Health Sciences College, Kotebe Metropolitan University, Addis Ababa, Ethiopia; 2 Department of Public health, College of Health Sciences, Arsi University, Assela, Ethiopia; 3 Department of Public Health Menelik II Medical and Health Sciences College, Kotebe Metropolitan University, Addis Ababa, Ethiopia

**Keywords:** Civil servant women, Addis Ababa, Ethiopia, Abdominal obesity, Waist circumference, Waist-hip ratio

## Abstract

**Background:**

Abdominal obesity increases the risk of cardio-metabolic diseases, disability, and poor quality of life, as well as health-care costs. It is a component of the metabolic syndrome, along with hypertension, diabetes, and dyslipidemia. The goal of this study was to determine the prevalence of abdominal obesity and associated risk factors among female civil servants in Addis Ababa, Ethiopia in 2021.

**Methods:**

An institution-based cross-sectional study was undertaken from March31^st^ to April 15^th^, 2021.A multi-stage sampling technique was employed to select 478 study participants. Data was entered into EpiData version 3.1 and then exported to SPSS version 21 for analysis. A descriptive data analysis was used to present the distribution of study variables. Bivariable and multivariable analyses were used to assess the relationship between independent variables and abdominal obesity at 95% CI. The level of statistical significance was declared at a *p*-value less than 0.05.

**Result:**

The prevalence of abdominal obesity defined by waist circumference was found to be29.5% (95% CI: 25.39-33.6%) and 32.8% (95% CI: 28.57%-37.03%) by waist hip ratio, respectively. Age group 29-37 years [AOR= 2.451, 95% CI: (1.199-5.013)], age group 38-46 years [AOR=3.807, 95% CI: (1.328-10.914)], age group 47-55 years [AOR=6.489, 95% CI: (1.367-30.805)], being married [AOR= 4.762, 95% CI: (2.321-9.721)],consumption of meat >=5 per week[ AOR= 4.764, 95% CI: (1.939-11.711)], having lunch daily[AOR= 0.388, 95% CI:(0.166-0.910)] and snack consumption [AOR=4.163, 95% CI:(1.503-11.534)] were significantly associated with abdominal obesity.

**Conclusion:**

The prevalence of abdominal obesity as measured by waist circumference and waist hip ratio was found to be moderate and high, respectively. Age, being married, high consumption of meat, and having lunch daily were identified as associated with abdominal obesity. Healthy diet health education and nutrition intervention should be considered, with a focus on married, meat-consuming, and older age-group female civil servants.

**Supplementary Information:**

The online version contains supplementary material available at 10.1186/s40795-022-00613-9.

## Background

Obesity is characterized as irregular or excessive fat accumulation that may affect health as a disorder of energy metabolism [1]. It is a significant public health concern in both developed and developing countries, affecting people of all ages and genders and related to a variety of chronic diseases such as type 2 diabetes, hypertension, coronary artery disease, and cancer [2–4]. According to 2016 data from the WHO, more than 1.9 billion adults aged 18 and older were overweight worldwide. Over 650 million of them were obese [5].

Abdominal obesity is especially linked to an increased risk of mortality, morbidity, disability, poor quality of life, and health care costs [6–9]. The prevalence of elevated waist circumference(WC) in adults has been demonstrated to be an independent risk factor for metabolic syndrome, like hypertension, diabetes mellitus, and cardiovascular diseases [10–12]. In adults, studies have reported a higher association between waist circumference and these anomalies than with body mass index (BMI) [13, 14].

Overweight, general obesity, abdominal obesity (AO), visceral fat obesity (VFO), and other forms of obesity are frequently classified using body mass index (BMI), waist circumference (WC), waist-to-hip ratio (WHR), skin fold thickness, and bio-impedance. BMI and waist circumference(WC) are the most commonly used measures [15]. Abdominal fat assessment is the best predictor of visceral fat, which is significantly linked with most metabolic abnormalities [16].

Waist circumference (WC) based indices have been described to assess body shape and fat distribution, especially abdominal visceral adiposity. The gold standard methods include computed tomography and magnetic resonance imaging [17, 18]. However, in a clinical or epidemiological setting, WC is the most often available measurement to determine visceral fat.

Despite the fact that studies on central obesity in Africa are few, recent research has revealed an unprecedented rise in central obesity prevalence [3]. According to WHO estimates, 1.2% of men and 6.0% of women in Ethiopia were overweight or obese in 2014 [19]. Between 1997 and 2016, the combined prevalence in the country grew considerably, from2.6 to 6.9% in females and from 0.6 to 1.9% in males [19].

In Ethiopia, a study conducted in 2015 reported that in urban areas, the prevalence of overweight and obesity was 12.1% and 2.8 percent, respectively [6]. In another recent study conducted in Woldia and urban areas of Northwest Ethiopia, the prevalence of central obesity among women was found to be 27.9% and 86.9%, respectively [20, 21].

From the data of the 2018 WHO, the non-communicable disease (NCD) country profiles of Ethiopia, it was estimated that the deaths from CVDs, cancers, diabetes, and other NCDs were 16%, 7%, 2%, and 12%, respectively, and the total estimated deaths from NCDs was 39% of all deaths [22].

According to the Ethiopian demographic and health survey (EDHS) 2016,the proportion of women who were overweight or obese had increased from 3% in 2000 to 8% in 2016, and the proportion of men who were obese was found to be 3% [23]. Even though there are no well documented national data and studies on central obesity, there are a few studies done on central obesity in different parts of Ethiopia, which revealed that the prevalence of central obesity is currently increasing. For instance, according to a study done in Dilla, Gonder, Dire dawa, and urban areas of Northwest Ethiopia, the prevalence of central obesity was found to be 24.4%, 33.6%, 46.6%, and 37.6%, respectively [21, 24–26]. Therefore, the aim of this study was to accurately assess the prevalence and risk factors associated with abdominal obesity in a volunteer sample of women employed in 2021 as civil servants in Addis Ababa, Ethiopia.

## Methods

### Study design, study area, and study period

An institution-based cross-sectional study design was conducted to assess the prevalence and associated risk factors of abdominal obesity among civil servant women in Addis Ababa city from March 31st to April 15th, 2021. In Addis Ababa city administration, the capital of Ethiopia, there are 11 sub-city and 117 district-level administration offices. Chartered in 1886, it has the status of both a state and a city. There are 13 public and 22 private hospitals, as well as 96 health centers in the capital.

### Study participants

Women working as civil servants in Addis Ababa city administration in different districts and willing to participate in the study were included. Women who have deformities around their hip and abdominal areas and who were temporary employees were excluded.

### Sample size determination

A single population proportion formula was used to calculate sample size considering 95% confidence level, 5% margin of error, and prevalence of central obesity from a study done in Dilla, Ethiopia at 27.3% [24]. Adding a 5% non-respondent rate and a 1.5% of design effect the final sample size was 478.

### Sampling technique and procedure

In this study, multi-stage random sampling was used. First, the three sub-cities were selected from 10 sub-cities by using a simple random sampling technique, which covered 30% of the total sub-cities of Addis Ababa. Kirkos, Yeka, and Bole sub-cities were selected as primary sampling units using simple random sampling. After selecting three sub-cities, three woredas were selected from each sub-city by simple random sampling as a secondary sampling unit. Then samples were allocated to each selected woreda proportionally based on their total number of civil servants. The list of civil servant workers from the selected districts was used as a sampling frame to select the participants of the study, by a simple random sampling technique, from each woreda (district).

### Data collection tools and procedures

An interviewer-administered structured questionnaire was adapted from WHO-stepwise for chronic non-communicable disease, having components of socio-demographic information; dietary intakes; physical activity; health risky behavior questions; and anthropometric measurement were made.

Participants were interviewed for their socio-demographic information, dietary intakes, physical activity, and health-risky behaviors. Anthropometric measurements were taken by trained professional nurses at the end of the interview. The food consumption habits of the participants were investigated by a semi-quantitative food frequency questionnaire (FFQ) by FAO and a food frequency questionnaire (FFQ) modified from the WHO-step-wise approach consisting of foods commonly consumed by the study population. Study subjects were asked to report their frequency of consumption and the number of times they consumed weekly [27, 28].

The global physical activity questionnaire (GPAQ) developed by WHO for physical activity surveillance was used to assess the physical activity pattern among selected individuals in three domains, including activity at work, travel to and from places, recreational activities, and sedentary behavior, through face-to-face interviews of the respondents in the study area. The level of total physical activity of study participants was classified as physically active (> 600 EM) or physically inactive (<600 EM) using the standard WHO total physical activity calculation guide [29].

Waist circumference was measured at the midpoint between the lower margin of the last palpable rib and the top of the iliac crest, using a stretch resistant tape that provides a constant 100 g tension. The hip circumference measurement was taken around the widest portion of the buttocks. The waist to hip ratio was calculated by dividing the waist circumference by the hip circumference. The study participants were instructed to wear light clothing and stand with their feet close together, arms at their sides, and body weight evenly distributed for both measurements. The subjects were asked to relax, and the measurements were taken at the end of a normal expiration.

The measurements were repeated twice, and when they were within 1 cm, the average was calculated. Otherwise, the two measurements were repeated. Measurement was taken before meals or three hours after meals. The measurement was also taken at the end of a gentle expiration, after taking a deep inhalation with the tape snug but not compressing the skin [30].

### Data quality control

Data quality was closely assured throughout the process, from tool development through to result analysis. First, the questionnaire was prepared in English and translated to the local language, Amharic. To maintain the tool's consistency, the Amharic text was then translated back into English. The research instrument which was used to measure abdominal obesity and associated risk factors was properly calibrated by defining each concept and assessing for content validity, in which the instrument items are adapted from the WHO-step-wise approach questionnaire found online by Google search and other standard questionnaires from FAO. To assess whether the instrument covered all dimensions of the construct, relevant literature and experts in the field were properly consulted. On the other hand, to maximize the quality of the data, data collectors were selected carefully based on their educational status. The training was also given on the nature and purpose of the research and the objective of the study.

### Data processing and analysis

After the data was collected, the collected data was checked for completeness and consistency manually. The data was edited, coded, and entered on to a statistical software package, Epi Data Version 3.1, and then was exported to IBM the Statistical Package for the Social Sciences Version 20 for analysis. Descriptive analysis was primarily used to show the distribution data from the research variables and to summarize it. Bivariate and multi-variable analyses were used to assess any relationship between each independent variable (socio-demographic characteristics, behavioral factors, dietary factors, and physical activity) and the outcome variable (abdominal obesity). Crude and adjusted odds ratios were used to ascertain any associations between the dependent and independent variables, while significance will be determined using 95% confidence intervals. Independent variables were found to be significant with a *p*-value less than 0.25 at the bi-variate level and were included in a multi-variable analysis. Finally, the results were presented in the frequency distribution table, chart, and graphs.

### Operational definitions

**Abdominal obesity**

**Abdominally obese**: WC>88 cm [30].

**Normal**: WC<=88 cm [30].

**Abdominally obese**: WHR*>=*0.85 [30].

**Normal**: WHR< 0.85 [30].

**Civil servant women**: are women employed by the Addis Ababa city government administration who are working at Woreda level.

**Woreda (District)**: is the third (lowest) level of the administrative division of Addis Ababa city administration, which was previously called “kebele.”

**Sub-city**: is the middle level of the administrative division of Addis Ababa city administration, which is called “Kifleketema” locally.

**Total physical activity metabolic equivalents (MET)-minutes/week**= the sum of the total metabolic equivalents (MET) minutes of activity computed for each setting.

**WHO total physical activity recommendations**; total physical activity MET minutes per week is greater than 600 [29].

**Total physical activity does not meeting WHO recommendations**: Total physical activity MET minutes per week is < 600 [29].

## Results

### Socio-demographic characteristics

From a total of 478 study participants, a complete set of information was obtained from 451 working civil servants, which gave a response rate of 94.4%.The mean and the standard deviation of the respondent age were 30.11 (±6.86) years, of which 55% of the respondents were between the age groups of 20-28 years. Among the study participants, 48.3% and 48.6% were single and married, respectively. More than half (68.1%) of the study participants were degree holders. Only 6% of the study participants had a master's degree or higher.

Of the participants’ Ethiopian Orthodox Tewahedo (81.6%) religion followers were the highest, followed by Protestants (12%) and Muslims (4.4%). The mean (SD) of the respondent’s salary was 5459.92(±2021.16) Ethiopian birr. 23.1% of the respondents got a monthly salary of less than 3934 Ethiopian birr. The mean family size of the respondents was 3.57, of which 56.8% of the respondents had a family size of four or above (Table 1).

**Table 1:** Demographic and socioeconomic characteristics of civil servant women in Addis Ababa city, Ethiopian, 2021 (*n* = 451)

**Variable**	**Frequency**	**Percent**
**Age**		
20-28	248	55.0
29-37	140	31.0
38-46	48	10.6
47-55	15	3.3
**Marital status**		
Single	218	48.3
Married	219	48.6
Divorced	14	3.1
**level of education**		
college diploma	117	25.9
Degree	307	68.1
masters and above	27	6
**Religion**		
Orthodox tewahido	368	81.6
Protestant	54	12.0
Muslim	20	4.4
Catholics9 2		
**Salary**		
<3934	104	23.1
3934-7070	192	42.6
>=7071	155	34.4
**Family size**		
<=3	195	43.2
>=4	256	56.8
**Age**		
20-28	248	55.0
29-37	140	31.0
38-46	48	10.6
47-55153.3		

### Factors influencing food consumption

According to the data obtained from food frequency, of the total respondents, 44.6% consumed fruit three or less times per month and 41.7% consumed it within a week. 53.2% of study participants consumed vegetables1-4 times per week. Regarding the consumption of bread and cereals, 41.5% were consumed daily and 58.5% were consumed less frequently. According to this data, cereals were the most common food group among respondents. More than half of the respondents (61.2%, 58.1%, 53.7, 69.2%, and 66.5%) consumed meat, legumes, milk products, fast food, and sweetened beverages three or less times a month, respectively (Table 2).

**Table 2:** Food consumption frequency among civil servant women in Addis Ababa city, Ethiopia, 2021 (*n*=451)

**Variable**	**Frequency**	**Percent**
**Fruit**		
three or less times monthly	201	44.6
1-4 per week	188	41.7
>=5 times per week	62	13.7
**Vegetables**		
three or less times monthly	112	24.8
1-4 times per week	247	54.8
>=5 times per week	92	20.4
**Bread and cereals**		
not daily	264	58.5
Daily	187	41.5
**Egg**		
less than once in a month	48	10.6
1-3 times monthly	140	31.0
>=5 times per week	263	58.3
**Meat**		
three or less times monthly	276	61.2
1-4 times per week	117	25.9
>=5 times per week	58	12.9
**Legumes**		
three or less times monthly	262	58.1
1-4 per week	135	29.9
>=5 times per week	54	12.0
**Milk, cheese, yogurt**		
three or less times monthly	242	53.7
1-4 times per week	155	34.4
>=5 times per week	54	12.0
**Sweets**		
three or less times monthly	209	46.3
1-4 times per week	129	28.6
>=5 times per week	113	25.1
**Fast food**		
three or less times monthly	312	69.2
1-4 times per week	108	23.9
>=5 times per week	31	6.9
**sweetened beverages**		
three or less times monthly	300	66.5
1-4 times per week	106	23.5
>=5 times per week	45	10.0

### Factors influencing dietary habits

Of the total respondents, 88.9% had three or more meals per day, and only 11.1% had fewer than three meals per day. Nearly three-fourths (70.1%)of the study participantsreported that they didn't consume breakfast on a daily basis. Only 29.9% of the respondentssaid theyconsumed breakfast on a daily basis.The majority of the respondents (88% and 86.7%) consumed lunch and dinner on a daily basis, respectively. More than two thirds (63.6%) of respondents commonly used seed oil (sunflower) for household food preparation,followed bypalm oil(28.4%) and butter(8%).Of the total study participants, 80.1% of the respondentsreported that they ate meals prepared at home on a daily basis (Table 3).

**Table 3:** Dietary habit among civil servant women in Addis Ababa city, Ethiopia 2021 (*n*=451)

**Variable**	**Frequency**	**Percent**
**Number of meals per day**		
<3 meal per day	50	11.1
>=3 meal per day	401	88.9
**Breakfast**		
not daily	54	70.1
daily	397	29.9
**lunch**		
not daily	54	12.0
daily	397	88.0
**Snack**		
no	311	69.0
yes	140	31.0
**Number of snacks**		
No	311	69
<=2 per day	101	22.4
>=3 per day39	8.6	
**dinner**		
not daily	60	13.3
Daily	391	86.7
**Eat during bed times**		
not daily	407	90.2
Daily	44	9.8
**meal out of home**		
never	88	19.5
not daily	329	72.9
Daily	34	7.5
**meal prepared at home**		
not daily	87	19.3
Daily	364	80.7
**Oil most used**		
seed oil	287	63.6
palm oil	128	28.4
Butter368.0		

### Behavioral factors

Of the total study participants, 98.7% were non-smokers. Nearly three-fourths (73.2) of the respondents had never consumed alcohol. About 96.2% of the respondents never chewed khat. Study participants who met the WHO recommendation fortotal physical activity level were 5.8%, and the rest (38.8%) didn’t meet the WHO recommendation. And the remaining 55.4% reported no participation in any physical activity (Table 4).

**Table 4:** Behavioral factor of civil servant women in Addis Ababa city, Ethiopia, 2021 (*n*=451)

**Variable**	**Frequency**	**Percent**
**Ever smoked tobacco**		
No	445	98.7
Yes	6	1.3
**current smoker**		
No	447	99.1
Yes	4	0.9
**No of cigarette sticks smoke in a day**		
No smokein a day	445	98.7
< 2 standard drinking	5	1.1
>=2 standard drinking	1	0.2
**Ever alcohol consumption**		
No	330	73.2
Yes	121	26.8
**number of standard drinks**		
No drink	332	73.6
< 2 standard drinking	40	8.9
>=2 standard drinking	79	17.5
Frequency of alcohol drink		
No drink	330	73.2
Daily	11	2.4
>=1 per week	77	17.1
<1 in a month	33	7.3
**khat chewing**		
No	434	96.2
Yes	17	3.8
**Total physical activity level**		
no physical activity	250	55.4
< 600MET(un meet)	175	38.8
>=600(meet)	26	5.8
**time spend sitting or reclining**		
< 5 hours	142	31.5
5-8 hours	309	68.5

### Prevalence of abdominal obesity

**Fig. 1:**
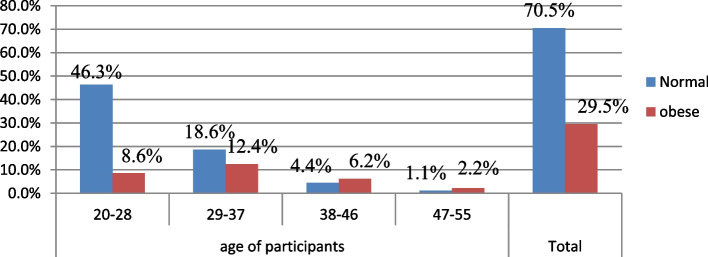
Prevalence of abdominal obesity by waist circumference among women civil servant working in Addis Ababa by age group Ethiopia, 2021

**Fig. 2:**
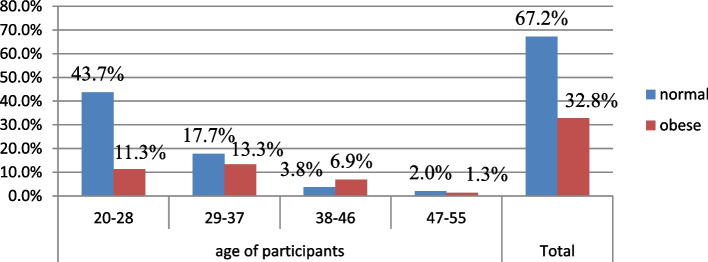
Prevalence of abdominal obesity by waist hip ratio among women civil servant working in Addis Ababa by age group Ethiopia, 2021

The prevalence of abdominal obesity among civil servant women working in Addis Ababa determined by waist circumference(WC) and waist-hip ratio (WHR) was 29.5%(95% CI:25.39%-33.61%) and 32.8% (95%CI:28.57%-37.03%) respectively. The prevalence of abdominal obesity was highest among the age groups of 27–38 by both waist circumference (12.4%) and waist-hip ratio (13.3%), respectively. The prevalence was lowest among the age group 47–55years, by both WC (2.3%) and WHR (1.3%) (Table 5, figs 1 and 2).

**Table 5:** Pervalence of abdominal obesity among civil servant women in Addis Ababa, Ethiopia, 2021 (*n*=451)

**Variable**	**Frequency**	**Percent**	**95%CI**
**Abdominal obesity by waist circumference (WC)**			
No	318	70.5	70.5 (66.3-74.7)
Yes	133	29.5	29.5 (25.3-33.7)
**Abdominal obesity bywaist to hip ratio(WHR)**			
No	303	67.2	67.2 (62.7-71.6)
Yes	148	32.8	32.8 (37.3-37.3

The mean (SD) of waist circumference among civil servant women working in Addis Ababa was 79.4(11.28) cm. Both the mean waist circumference and waist hip ratio among civil servant women were slightly lower than the WHO cut-off points.

## Associated Risk Factors for Abdominal Obesity by Waist Circumference

### Multivariate analysis

In multi-viariable logistic regression analysis, variables with a *p*-value less than 0.25 from bi-variable analysis were included. Based on this criteria, age, marital status, consumption of meat, consumption of snacks, and having lunch daily were significantly associated with abdominal obesity at a *p*-value less than 0.05. 29–37-year-olds, 38–46-year-olds, and 47–55-year-olds were 2.451 [(AOR=2.451, 95% CI: (1.199-5.013), 3.807 (AOR=3.807, 95% CI: (1.328-10.914), and 6.489 (AOR=6.489, 95% CI: (1.367-30.805) times more likely to develop abdominal obesity than 20–28-year-olds, respectively.

Being married was 4.762 times more likely to develop abdominal obesity [AOR=4.762;95% CI: (2.321-9.771)] than unmarried women. Respondents who consumed meat more or equal to five times per week were found to be 4.764 more likely to develop abdominal obesity[AOR=4.764; 95% CI= (1.939-11.711)] as compared to those who consumed meat three or less times per month. Having lunch every day reduced the risk of developing abdominal obesity by 61.2% [AOR = 0.388, 95% CI: (0.166-0.910)]. Respondents who consumed snacks were 4.163 times more likely to be abdominally obese than those who did not consume snacks [AOR= 4.163; 95% CI:(1.503-11.534)] (Table 6).

**Table 6:** Multi logistic regression of factors associated with abdominal obesity by waist circumference among civil servant women in Addis Ababa, Ethiopia, 2021 (*n*=451)

	**abdominal obesity by WC**		**Odd ratio (CI 95%)**	
**Variable**	**Yes(obese)**	**No(normal)**	**COR**	**AOR**
**Age**				
20-28	39	209	1	**1**
29-37	56	84	3.57(2.20-5.77)*	2.451(1.199-5.013) *
38-46	28	20	7.50(3.8-14.63)*	3.807(1.328-10.914)*
47-55	10	5	10.718(3.474-33.68)*	6.489(1.367-30.805)*
**Marital status**				
Single	28	190	1	**1**
Married	101	118	5.808(3.603- 9.363)*	4.762(2.321-9.771)*
Divorced	4	10	2.714(0.797-9.245)	0.686(0.139-3.394)
**Religion**				
Orthodox tewahido	108	260	1	1
Protestant	19	35	1.30(0.716-2.386)	1.893( 0.822-4.362)
Muslim	3	17	0.425(0.122-1.479)	.610(0.126-2.961)
Catholics	3	3	2.407(0.478-12.116)	5.070(0.460-55.941)
**Salary**				
<3934	19	85	1	**1**
3934-7070	48	144	1.49(0.822-2.704)	1.093(0.497-2.404)
>=7071	66	89	3.318(1.83-5.98)*	1.192(0.500-2.844)
**Family size**				
<=3	39	156	0.431(0.279-0.664)*	0.959(0.531-1.732)
>=4	94	162	1	1
**Bread and cereals**				
not daily	71	193	1	1
once or more times per day	62	125	1.348(0.896-2.028)	1.031(0.582-1.825)
**Fruit**				
three or less times monthly	49	152	0.677(0.363-1.261)	0.994(0.405-2.439)
1-4 per week	64	124	1.084(0.588-1.99)	1.221(0.510-2.921)
>=5 times per week	20	42	1	1
**Meat**				
three or less times monthly	56	220	1	**1**
1-4 times per week	47	70	2.638(1.646-4.228)*	2.287(1.209- 4.325) *
>=5 times per week	30	28	4.209(2.327-7.614)*	4.764(1.939-11.711)*
**Legumes**				
three or less times monthly	68	194	1	1
1-4 per week	47	88	1.524(0.972-2.388)	.890(0.312-1.654)
>=5 times per week	18	36	1.426(0.76-2.677)	.885(0.362-2.163)
**Milk, cheese, yogurt**				
three or less times monthly	64	178	1	1
1-4 times per week	47	108	1.210(0.775-1.891)	.607(0.312-1.181)
>=5 times per week	22	32	1.912(1.035-3.531)*	.768(0.308-1.915)
**Sweets**				
three or less times monthly	48	161	1	1
1-4 times per week	51	78	2.193(1.360-3.537)*	1.937(0.987-3.802)
>=5 times per week	34	79	1.444(0.862-2.417)	1.923(0.888-3.940)
**sweetened beverages**				
three or less times monthly	82	218	1	**1**
1-4 times per week	30	76	1.049(0.641- 1.718)	.751(0.357-1.578)
>=5 times per week	21	24	2.326(1.229- 4.404)*	1.844(0.712-4.776)
**Number of meal per day**				
<3 meal per day	10	40	1	1
>=3 meal per day	123	278	1.770(0.857- 3.653)	1.137(0.451-2.866)
**Lunch**				
not daily	21	33	1	1
Daily	112	112	0.618(0.343-1.113)	0.388(0.166-0.910)*
**meal prepared at home**				
not daily	19	68	0.613(0.352- 1.062)	0.837(0.375-1.867)
Daily	114	250	1	
**Snack**				
No	81	230	1	1
Yes	52	88	1.678(1.096- 2.570)*	4.163(1.503-11.534)*
**Frequency of alcohol drink**				
No drink	75	255	1	1
Daily	5	6	1.414(0.404-4.940)	0.923(0.152-5.602)
>=1 per week	42	35	2.539(1.530- 4.212)*	2.426(0.768-7.663)
<1 in a month	11	22	1.076(0.493-2.346)	NA
**time spend sitting or reclining**				
< 5 hours	29	113	1	1
5-8 hours	104	205	1.977(1.234- 3.167)*	1.087(0.580-2.039)

*AOR* Stands for "Adjusted odds ratio", *COR* Stands for "Crude odds ratio"

^*^Statistically significant variables with a *p*-value of less than 0.05; 1-reference group

## Factors associated with abdominal obesity by waist-to-hip ratio

### Multivariate analysis

In a multivariate analysis for abdominal obesity measured by waist hip ratio, age, marital status (being married) and consumption of snacks were variables associated with abdominal obesity. Women who were married were 2.448 times more likely to develop abdominal obesity than those who were single. Similarly, the respondent age groups of 29–37 and 38–46 years were 2.637 [AOR = 2.637; 95% CI: (1.452–4.788)] and 5.439 [AOR = 5.439; 95% CI: (2.277–12.995)] times more likely to develop abdominal obesity than the age group of 20–28 years, respectively. The respondents who consumed snacks were 3.270 times more likely to develop abdominal obesity than those who didn’t consume snacks [AOR = 3.270; 95% CI: (1.437–7.442)] (Table 7).

**Table 7:** Multivariable logistic regression analysis of factors associated with abdominal obesity by waist hip ratio among civil servant women in Addis Ababa city, Ethiopia 2021 (*n*=451)

	**abdominal obesity by WHR**		**Odd ratio( CI 95%)**	
**Variable**	**Yes(obese)**	**No(normal)**	**COR**	**AOR**
**Age**				
20-28	51	197	1	1
29-37	60	80	2.897(1.838-4.565)*	2.637(1.452- 4.788)*
38-46	3	17	7.044(3.616-13.722)*	5.439(2.277-12.995)*
47-55	6	9	2.575(0.876-7.567)	1.405(0.400- 4.938)
**Marital status**				
Single	41	177	1	1
Married	103	116	3.833(2.491-5.899)*	2.448(1.394-4.299) *
Divorced	4	10	1.727(0.516-5.781)	.591(0.148-2.121)
**Salary**				
<3934	30	74	1	1
3934-7070	54	138	0.965(0.569-1.637)	.593(0.324-1.087)
>=7071	64	91	1.735(1.020-2.951)*	.583(.299-1.138)
**Family size**				
<=3	51	144	0.581(0.386-0.872)*	1.070(0.653-1.753)
>=4	97	159	1	1
**Meat**				
three or less times monthly	75	201	1	1
1-4 times per week	46	71	1.736(1.101-2.740)*	1.379(0.814-2.336)
>=5 times per week	27	31	2.334(1.307-4.169)*	1.841(0.886-3.828)
**Milk, cheese, yogurt**				
three or less times monthly	72	170	1	1
1-4 times per week	54	101	1.262(0.821-1.941)	0.929(0.553-1.562)
>=5 times per week	22	32	1.623(0.883-2.984)	1.028(0.488-2.168)
**Sugar and Sweets**				
three or less times monthly	60	149	1	1
1-4 times per week	50	79	1.572(0.988-2.500)	1.220(0.704-2.113)
>=5 times per week	38	75	1.258(0.769-2.058)	1.370(0.765-2.453)
**Number of meals per day**				
<3 meal per day	10	40	1	1
>=3 meal per day	138	263	2.099(1.019-4.325)*	1.568(0.706-3.481)
**Snack**				
No	91	220	1	1
Yes	57	83	1.660(1.095-2.518)*	3.270(1.437-7.442) *
**Meal prepared at home**				
not daily	24	63	0.737(0.439-1.237)	1.392(0.733-2.641)
Daily	124	240	1	1
**Oil most used**				
seed oil	95	192	1	
palm oil	45	83	1.096(0.707-1.698)	1.411(0.823- 2.418)
Butter	8	28	0.577(0.253-1.315)	0.656(0.260-1.654)
**Total physical activity level**				
no physical activity	93	157	3.258(1.089-9.746)*	2.929(0.845-10.146)
< 600(met)	51	124	2.262(0.742-6.892)	1.875(0.540-6.515)
>=600(met)	4	22	1	1

^*^P-value at<0.05

## Discussion

This study aimed to determine the prevalence and associated risk factors for abdominal obesity among civil servant women in Addis Ababa. The overall magnitude of abdominal obesity as defined by waist circumference and waist hip ratio among civil servant women was found to be 29.5% and 32.8%, respectively. This result was slightly greater than the studies done in Dilla based on WC (27.3%) [24] and Woldia Town based on both WC and WHR (24.3%, 27.9%) [20] and also higher than the study in Addis Ababa among working adultsbased on WC(19.6%) [31]. The possible explanation for the differencecould be the fact that a difference in place, setting, or time setting could influence, variation in WC and WHR cut off points,as well as may be for sociodemographic disparitiest. The prevalence of abdominal obesity in this study was high when compared to findings from Dilla and Woldia.This might be due to the lifestyle changes in Addis Ababa,the sedentary behavior of civil servants, and the nutrition transition adopted from western countries.

A study conducted in Ghana among female teachers revealed that the prevalence of abdominal obesity defined by WHR and WC was found to be 17.8% and 59% [32] respectively, which was not consistent with the current study prevalence of abdominal obesity defined by WHR (32.8%) and WC (29.5%).The difference in these results might be due to the study period and WHR and WC cut-off points. A study done in Ghana considered WC >80 cm, while this study considered WC >88cm.The study conducted in Ghana considered WHR > 0.85, but this study considered abdominal obesity > 0.85. The other possible explanation for the difference could be socio-demographic factors and dietary patterns.

The prevalence of abdominal obesity (85.9%) defined by WC in northwesturban areas of Ethiopia [21] among women was much higher when compared to the current study(29.5%).Contrariwise, the current finding was higher than in a study conducted in Nigeria among civil servants (23.1%) [33] and yet lower than the study done in Russia (44%) [34] among bank employees. The difference could be study period, WHO cut-off point variation, socioeconomic difference, socio-demographic factorsand dietary intake pattern or differences in MBI among population groups.

The prevalence of abdominal obesity by WC in Gaza Strip-Palestine was 82.2%, which was very high when compared to the current study of abdominal obesity by WC. The difference might be due to WHO cut-off pointsand socio-cultural and economic differences [35]. The study findings of Gaza Strip-Palestine considered WC >80 cm, but the current study considerswaist circumference(WC) greater than 88 cm. The other possible explanation for the difference might be the type of food consumed, socio-economic status, or age of the study participants; the current study considered age from 20 and the study in Palestine starts at greater or equal to 26 years.

Likewise, a study done in Panama among women showed the highest prevalence of abdominal obesity, which was reported by WC (97.9%), which was three times that of the current study [36]. This might be because Panamanian women consume beverages or sugar-rich foods and have socio-demographic characteristics leading to higher BMI. Sugary beverages or foods were statistically associated with abdominal obesity among Panamanian women. Similarly, the prevalence of abdominal obesity in studies conducted in Indonesia (68.3%) among adult female employees was higher than in the current study, but a study conducted in Iran (34.6%) among adult females was almost consistent with this study [37, 38].

This study revealed that, the odds of being abdominally obese by WC increased by age. The age groups of 29–37, 38–46, and 47–55 years were 2.553, 4.027, and 7.008 times more likely to develop abdominal obesity, respectively, as compared to the age group 20–29. This result was consistent with a study conducted in Nigeria among civil servants and in southern America [39, 40]. This might be due to sex hormone changes and a decrease in physical activity levels with ageing. The findings of this study also revealed that the age groups 29–37 and 38–46 years were significantly associated with AO defined by WHR, but the age group 47–55 years was not associated with AO, unlike that of WC. Thus, this finding was inconsistent with the finding from Ghana [32].

Marital status was one of the predictors of abdominal obesity among civil servant women in this study. Consistent with studies conducted in Greece, Nigeria, and Iran [41–43]. This can be explained by the fact that women after marriage may have less physical activity, changed dietary patterns, and experience pregnancy-induced social support. Married women have more social support than those who are not married. This marital support can lead to obesity through food, activity, and social values. Some people control their weight to attract mates, and once they get married, weight control may be less valued, so that diet/exercise behaviors for slimness may be neglected or they may not give attention to attractiveness once they have gotten married and experienced pregnancy, nursing and the stress of family life [43].

The findings of this study revealed that consumption of meat more than even once a week was associated with abdominal obesity by WC. The more meat consumed the greater were the odds of developing abdominal obesity . The OR for eating meat 1-4 times a week was 2.342 compared to those who ate meat three or fewer times a month. Likewise, using meat products morethan or equal to five times perweek increased the OR for abdominal obesity to 5.257. Because a possible explanation is that meat has high energy and high fat content that might be associated with a higher risk of being overweight, including general and central obesity [44]. This result was similar to studies done in the USA ,Woldia, and Hawassa [20, 45, 46].

Having lunch daily was significantly associated alower risk for abdominal obesity in this study. Theincidence of abdominal obesity was 61.2% lower in those who had lunch daily than only occasionally. Findings from China also showed that skipping lunch was positively associated with obesity in women [47]. In general, findings from various studies have confirmed that meals skipped are associated with overweight, obesity, and abdominal obesity [13, 48, 49]. It might be due to the decreased thermic effect of food after an irregular meal pattern when compared with individuals with a regular meal pattern. The reduced thermic effect with irregular meal frequency may lead to weight gain in the long term. On the other hand restrictive eating is being studied extensively as a weight loss and longetivity strategy [50].

This study revealed that those who consumed snacks were 4.163 and 3.270 times more likely to develop abdominal obesity as measured by WC and WHR respectively than those who didn’t consume snacks. Although, the relationship between snacking and obesity or abdominal obesity is unclear, some studies have suggested that consumption of energy-dense, high-sugar, high-fat snacks is a key factor in obesity [51]. Other studies failed to establish a relationship between snacking and obesity or abdominal obesity [52]. This may be because the type of snack eaten matters. Those who eat energy-dense and sugary snacks may be especially susceptible to abdominal obesity, as opposed to those who eat healthy snacks. This result is supported by studies conducted in association with South East Asian Nations countries and northeast Ethiopia [53, 54].

## Limitation

This study could have some limitations most notably that height and weight were not measured which affect the results directly or indirectly. Some of the limitations emanate from the nature of the cross-sectional study since the outcome (abdominal obesity) and predictor variables relationships were temporal and examined at the same time, therefore no causal deduction can be made. The study did not include other measurements like skin fold thickness. The portion size of food consumed by respondents was not assessed. The type of snack they were practicing was not identified. On the other hand, there might be over and underestimations of food frequency and meal habits, alcohol consumption, physical exercises, and time spent sitting and reclining due to recall bias.

## Conclusion

A relatively high 29.5% and 32.8% of the study participants were found to be centrally obese based on abdominal obesity defined by WC and WHR. Age groups of 29–37 years, 38–46 years, and 47–55 years, being married, meat consumption 1-4 days and >=5 days, having lunch daily, and snack consumption were the predictors of abdominal obesity based on WC in this study of women civil servant. Age groups of 29–37 years and 38–46 years, snack consumption, and being married were the predictors of abdominal obesity based on WHR in this study of women civil servant employees in Addis Ababa. Governmental and non-governmental organizations should provide special awareness campaigns regarding abdominal obesity for married and older age group civil servant women in collaboration with other stakeholders like the city-administration of women’s affairs and Addis Ababa city-administration health Bureau. Regular health education should be considered for female civil servants regarding the frequency of meat consumption and of unhealthy snacks.

## Supplementary Information

**Additional file 1.**

## Data Availability

All data generated or analyzed during this study are included in this published article.

## Abbreviations

AO: Abdominal Obesity
BMI: Body Mass Index
CI: Confidence Interval
CHO: Carbohydrate
CVD: cardiovascular disease
DDS: Dietary Diversity Score
FVS: Food Variety Scores
HEI: Health Eating Index
METs: Metabolic Equivalents
NCD: Noncommunicable Diseases
GDP: Gross Domestic Product
NHLBI: National Heart, Lung, and Blood Institute
SPSS: Statistical Package for Social Science
WHR: Waist Hip Ratio
WHO: World Health Organization
VAI: Visceral Adiposity Index
WHtR: Waist-to-Height Ratio
