# Supplementary material for: Prevalence of abdominal obesity and associated risk factors among women civil servants in Addis Ababa, Ethiopia, 2021: an institution-based study
Source: BMC Nutr. 2022 Oct 24;8:119. doi: 10.1186/s40795-022-00613-9 (PMC9589629; doi:10.1186/s40795-022-00613-9)
Supplement: Supplementary file 1 — Additional file 1. [file 40795_2022_613_MOESM1_ESM.pdf]

Sheet11650871923564

T	SUGRSWET	SWEETBVG	FASTFOOD	NOFMEALS	BREKFAST	LUNCHINT	SNACKDOY	NOFSNACK	DINNERIN	EATBEDTM	MEALOTHM	HOMMMEA
	three or less times monthly	three or less times monthly	three or less times monthly	>=3 meal per day	not daily	daily	no	no snack	daily	not daily	daily	daily
	>=5 times per week	>=5 times per week	three or less times monthly	>=3 meal per day	daily	daily	no	no snack	daily	not daily	daily	not daily
	>=5 times per week	1-4 times per week	1-4 times per week	>=3 meal per day	daily	daily	no	no snack	daily	not daily	not daily	daily
	three or less times monthly	1-4 times per week	1-4 times per week	>=3 meal per day	daily	daily	no	no snack	daily	not daily	not daily	daily
	1-4 times per week	three or less times monthly	three or less times monthly	>=3 meal per day	not daily	daily	no	no snack	daily	not daily	not daily	daily
	three or less times monthly	three or less times monthly	three or less times monthly	>=3 meal per day	not daily	daily	no	no snack	daily	not daily	not daily	daily
	1-4 times per week	1-4 times per week	1-4 times per week	>=3 meal per day	not daily	daily	no	no snack	daily	not daily	never	daily
	>=5 times per week	1-4 times per week	>=5 times per week	<3 meal per day	not daily	not daily	no	no snack	daily	not daily	not daily	daily
	three or less times monthly	three or less times monthly	three or less times monthly	<3 meal per day	not daily	daily	no	no snack	daily	not daily	never	daily
	three or less times monthly	three or less times monthly	three or less times monthly	>=3 meal per day	not daily	daily	no	no snack	daily	not daily	not daily	not daily
	three or less times monthly	three or less times monthly	three or less times monthly	>=3 meal per day	daily	daily	no	no snack	daily	not daily	never	daily
	three or less times monthly	three or less times monthly	three or less times monthly	>=3 meal per day	not daily	daily	no	no snack	daily	not daily	not daily	not daily
	>=5 times per week	1-4 times per week	three or less times monthly	>=3 meal per day	not daily	daily	no	no snack	daily	not daily	not daily	not daily

three or less times monthly	three or less times monthly	1-4 times per week	>=3 meal per day	not daily	daily	no	no snack	daily	not daily	not daily	not daily
three or less times monthly	three or less times monthly	1-4 times per week	>=3 meal per day	not daily	daily	no	no snack	daily	not daily	not daily	daily
three or less times monthly	three or less times monthly	three or less times monthly	<3 meal per day	not daily	not daily	no	no snack	daily	not daily	never	daily
1-4 times per week	three or less times monthly	three or less times monthly	>=3 meal per day	not daily	daily	no	no snack	daily	daily	not daily	not daily
1-4 times per week	three or less times monthly	three or less times monthly	>=3 meal per day	daily	daily	no	no snack	daily	not daily	not daily	daily
three or less times monthly	>=5 times per week	1-4 times per week	>=3 meal per day	not daily	not daily	no	no snack	daily	not daily	not daily	daily
>=5 times per week	three or less times monthly	three or less times monthly	>=3 meal per day	daily	daily	no	no snack	not daily	not daily	daily	daily
three or less times monthly	1-4 times per week	three or less times monthly	<3 meal per day	not daily	daily	no	no snack	not daily	not daily	not daily	not daily
three or less times monthly	three or less times monthly	three or less times monthly	>=3 meal per day	daily	daily	no	no snack	daily	not daily	not daily	daily
>=5 times per week	three or less times monthly	three or less times monthly	>=3 meal per day	daily	daily	no	no snack	daily	not daily	not daily	daily
three or less times monthly	three or less times monthly	three or less times monthly	>=3 meal per day	not daily	daily	no	no snack	daily	not daily	not daily	daily
1-4 times per week	1-4 times per week	1-4 times per week	<3 meal per day	daily	daily	no	no snack	daily	not daily	not daily	not daily
>=5 times per week	three or less times monthly	1-4 times per week	>=3 meal per day	not daily	not daily	no	no snack	not daily	not daily	not daily	daily
>=5 times per week	three or less times monthly	three or less times monthly	<3 meal per day	not daily	daily	no	no snack	daily	not daily	never	daily
>=5 times per week	1-4 times per week	1-4 times per week	<3 meal per day	not daily	daily	no	no snack	daily	not daily	never	daily

1-4 times per week	three or less times monthly	three or less times monthly	<3 meal per day	not daily	daily	no	no snack	daily	not daily	not daily	daily
three or less times monthly	1-4 times per week	three or less times monthly	>=3 meal per day	not daily	daily	no	no snack	daily	not daily	never	daily
>=5 times per week	three or less times monthly	three or less times monthly	<3 meal per day	daily	not daily	no	no snack	daily	not daily	not daily	not daily
three or less times monthly	three or less times monthly	three or less times monthly	>=3 meal per day	not daily	daily	no	no snack	daily	not daily	not daily	daily
three or less times monthly	1-4 times per week	1-4 times per week	>=3 meal per day	not daily	daily	no	no snack	daily	not daily	daily	not daily
three or less times monthly	three or less times monthly	three or less times monthly	>=3 meal per day	not daily	daily	no	no snack	daily	not daily	not daily	daily
three or less times monthly	three or less times monthly	three or less times monthly	>=3 meal per day	daily	daily	no	no snack	daily	not daily	not daily	daily
three or less times monthly	three or less times monthly	1-4 times per week	>=3 meal per day	not daily	daily	no	no snack	daily	not daily	not daily	daily
three or less times monthly	three or less times monthly	three or less times monthly	<3 meal per day	daily	daily	no	no snack	daily	not daily	not daily	daily
three or less times monthly	three or less times monthly	three or less times monthly	>=3 meal per day	daily	not daily	no	no snack	daily	not daily	not daily	daily
three or less times monthly	three or less times monthly	three or less times monthly	>=3 meal per day	not daily	daily	no	no snack	not daily	not daily	not daily	daily
three or less times monthly	1-4 times per week	three or less times monthly	>=3 meal per day	daily	daily	no	no snack	daily	not daily	not daily	daily
three or less times monthly	1-4 times per week	three or less times monthly	>=3 meal per day	not daily	daily	no	no snack	daily	not daily	never	daily
three or less times monthly	1-4 times per week	three or less times monthly	<3 meal per day	not daily	not daily	no	no snack	daily	not daily	daily	not daily

1-4 times per week	>=5 times per week	three or less times monthly	>=3 meal per day	daily	daily	no	no snack	daily	not daily	not daily	daily
three or less times monthly	three or less times monthly	three or less times monthly	>=3 meal per day	daily	not daily	no	no snack	not daily	daily	never	daily
1-4 times per week	three or less times monthly	three or less times monthly	>=3 meal per day	not daily	daily	no	no snack	daily	not daily	not daily	daily
1-4 times per week	1-4 times per week	1-4 times per week	>=3 meal per day	not daily	daily	no	no snack	daily	not daily	not daily	not daily
three or less times monthly	three or less times monthly	three or less times monthly	>=3 meal per day	not daily	daily	no	no snack	daily	not daily	not daily	daily
three or less times monthly	three or less times monthly	three or less times monthly	<3 meal per day	not daily	daily	no	no snack	daily	not daily	never	daily
1-4 times per week	three or less times monthly	three or less times monthly	>=3 meal per day	daily	daily	no	no snack	daily	not daily	not daily	daily
three or less times monthly	>=5 times per week	three or less times monthly	>=3 meal per day	not daily	daily	no	no snack	daily	daily	not daily	daily
three or less times monthly	three or less times monthly	>=5 times per week	>=3 meal per day	daily	daily	no	no snack	daily	not daily	not daily	not daily
three or less times monthly	three or less times monthly	three or less times monthly	>=3 meal per day	not daily	daily	no	no snack	daily	not daily	not daily	daily
1-4 times per week	three or less times monthly	three or less times monthly	>=3 meal per day	not daily	daily	no	no snack	daily	not daily	never	daily
1-4 times per week	three or less times monthly	three or less times monthly	>=3 meal per day	not daily	daily	no	no snack	daily	not daily	not daily	daily
three or less times monthly	three or less times monthly	three or less times monthly	>=3 meal per day	daily	daily	no	no snack	not daily	not daily	never	daily
three or less times monthly	three or less times monthly	three or less times monthly	>=3 meal per day	not daily	daily	no	no snack	daily	not daily	never	daily

>=5 times per week	>=5 times per week	three or less times monthly	>=3 meal per day	not daily	daily	no	no snack	daily	not daily	not daily	daily
1-4 times per week	three or less times monthly	three or less times monthly	>=3 meal per day	not daily	daily	no	no snack	daily	not daily	not daily	daily
>=5 times per week	three or less times monthly	>=5 times per week	>=3 meal per day	not daily	daily	no	no snack	daily	not daily	never	daily
three or less times monthly	three or less times monthly	three or less times monthly	>=3 meal per day	not daily	daily	no	no snack	daily	not daily	not daily	daily
three or less times monthly	three or less times monthly	three or less times monthly	>=3 meal per day	not daily	daily	no	no snack	daily	not daily	not daily	daily
1-4 times per week	three or less times monthly	three or less times monthly	>=3 meal per day	not daily	daily	no	no snack	daily	not daily	not daily	daily
three or less times monthly	three or less times monthly	three or less times monthly	>=3 meal per day	daily	daily	no	no snack	daily	not daily	daily	not daily
three or less times monthly	three or less times monthly	three or less times monthly	>=3 meal per day	not daily	daily	no	no snack	daily	not daily	not daily	daily
>=5 times per week	three or less times monthly	three or less times monthly	>=3 meal per day	not daily	daily	no	no snack	daily	not daily	not daily	not daily
three or less times monthly	three or less times monthly	three or less times monthly	>=3 meal per day	daily	not daily	no	no snack	daily	daily	not daily	not daily
>=5 times per week	>=5 times per week	>=5 times per week	<3 meal per day	not daily	daily	no	no snack	daily	not daily	not daily	daily
three or less times monthly	three or less times monthly	three or less times monthly	>=3 meal per day	not daily	daily	no	no snack	daily	not daily	not daily	daily
>=5 times per week	1-4 times per week	1-4 times per week	>=3 meal per day	not daily	daily	no	no snack	daily	not daily	not daily	daily
>=5 times per week	1-4 times per week	three or less times monthly	>=3 meal per day	not daily	daily	no	no snack	daily	not daily	not daily	daily

>=5 times per week	three or less times monthly	>=5 times per week	>=3 meal per day	not daily	not daily	no	no snack	daily	not daily	not daily	daily
>=5 times per week	three or less times monthly	three or less times monthly	>=3 meal per day	not daily	daily	no	no snack	daily	not daily	not daily	daily
three or less times monthly	three or less times monthly	three or less times monthly	>=3 meal per day	not daily	daily	no	no snack	daily	not daily	not daily	daily
1-4 times per week	1-4 times per week	three or less times monthly	>=3 meal per day	not daily	not daily	no	no snack	daily	not daily	not daily	daily
three or less times monthly	three or less times monthly	1-4 times per week	<3 meal per day	daily	daily	no	no snack	daily	not daily	daily	not daily
1-4 times per week	1-4 times per week	1-4 times per week	>=3 meal per day	not daily	daily	no	no snack	daily	not daily	not daily	daily
>=5 times per week	three or less times monthly	three or less times monthly	>=3 meal per day	not daily	daily	no	no snack	daily	not daily	not daily	not daily
three or less times monthly	1-4 times per week	1-4 times per week	>=3 meal per day	daily	daily	no	no snack	daily	not daily	not daily	daily
>=5 times per week	three or less times monthly	three or less times monthly	>=3 meal per day	daily	daily	no	no snack	daily	not daily	not daily	daily
>=5 times per week	three or less times monthly	1-4 times per week	<3 meal per day	daily	daily	no	no snack	daily	not daily	not daily	daily
three or less times monthly	1-4 times per week	1-4 times per week	>=3 meal per day	not daily	daily	no	no snack	daily	not daily	never	daily
three or less times monthly	1-4 times per week	1-4 times per week	>=3 meal per day	not daily	daily	no	no snack	daily	not daily	not daily	daily
>=5 times per week	1-4 times per week	three or less times monthly	>=3 meal per day	not daily	daily	no	no snack	not daily	not daily	not daily	not daily
three or less times monthly	three or less times monthly	three or less times monthly	>=3 meal per day	not daily	daily	no	no snack	daily	not daily	not daily	daily

three or less times monthly	three or less times monthly	three or less times monthly	>=3 meal per day	not daily	not daily	no	no snack	not daily	not daily	not daily	not daily
three or less times monthly	1-4 times per week	three or less times monthly	>=3 meal per day	not daily	not daily	no	no snack	not daily	not daily	daily	not daily
three or less times monthly	three or less times monthly	three or less times monthly	>=3 meal per day	daily	daily	no	no snack	daily	not daily	not daily	daily
1-4 times per week	>=5 times per week	>=5 times per week	<3 meal per day	not daily	daily	no	no snack	daily	not daily	not daily	daily
three or less times monthly	three or less times monthly	three or less times monthly	>=3 meal per day	not daily	daily	no	no snack	daily	not daily	not daily	daily
three or less times monthly	three or less times monthly	1-4 times per week	<3 meal per day	daily	daily	no	no snack	daily	daily	not daily	daily
three or less times monthly	three or less times monthly	three or less times monthly	>=3 meal per day	not daily	daily	no	no snack	daily	not daily	never	daily
three or less times monthly	three or less times monthly	three or less times monthly	>=3 meal per day	not daily	daily	no	no snack	daily	not daily	not daily	daily
>=5 times per week	three or less times monthly	1-4 times per week	>=3 meal per day	daily	daily	no	no snack	daily	not daily	not daily	daily
1-4 times per week	1-4 times per week	1-4 times per week	>=3 meal per day	daily	daily	no	no snack	daily	not daily	not daily	not daily
three or less times monthly	three or less times monthly	>=5 times per week	>=3 meal per day	not daily	daily	no	no snack	daily	not daily	not daily	daily
>=5 times per week	three or less times monthly	1-4 times per week	>=3 meal per day	not daily	daily	no	no snack	daily	not daily	never	daily
>=5 times per week	>=5 times per week	>=5 times per week	>=3 meal per day	not daily	daily	no	no snack	daily	daily	not daily	not daily
1-4 times per week	three or less times monthly	three or less times monthly	>=3 meal per day	not daily	daily	no	no snack	daily	not daily	not daily	daily

1-4 times per week	three or less times monthly	three or less times monthly	>=3 meal per day	not daily	daily	no	no snack	daily	not daily	not daily	not daily
>=5 times per week	three or less times monthly	>=5 times per week	>=3 meal per day	not daily	daily	no	no snack	daily	not daily	daily	not daily
>=5 times per week	three or less times monthly	1-4 times per week	>=3 meal per day	daily	daily	no	no snack	daily	not daily	daily	daily
three or less times monthly	three or less times monthly	three or less times monthly	>=3 meal per day	not daily	daily	no	no snack	daily	not daily	not daily	not daily
1-4 times per week	three or less times monthly	1-4 times per week	>=3 meal per day	not daily	daily	no	no snack	daily	not daily	not daily	daily
three or less times monthly	three or less times monthly	three or less times monthly	>=3 meal per day	not daily	daily	no	no snack	daily	not daily	not daily	daily
three or less times monthly	three or less times monthly	three or less times monthly	>=3 meal per day	not daily	daily	no	no snack	daily	not daily	not daily	daily
1-4 times per week	1-4 times per week	three or less times monthly	<3 meal per day	daily	not daily	no	no snack	not daily	not daily	not daily	daily
1-4 times per week	>=5 times per week	three or less times monthly	>=3 meal per day	not daily	daily	no	no snack	daily	not daily	not daily	daily
three or less times monthly	three or less times monthly	three or less times monthly	>=3 meal per day	not daily	daily	no	no snack	daily	not daily	not daily	daily
>=5 times per week	1-4 times per week	>=5 times per week	>=3 meal per day	daily	not daily	no	no snack	not daily	daily	never	not daily
three or less times monthly	three or less times monthly	three or less times monthly	>=3 meal per day	not daily	daily	no	no snack	daily	not daily	not daily	daily
1-4 times per week	1-4 times per week	1-4 times per week	<3 meal per day	not daily	daily	no	no snack	not daily	not daily	not daily	daily
1-4 times per week	three or less times monthly	1-4 times per week	>=3 meal per day	not daily	daily	no	no snack	daily	not daily	not daily	daily

>=5 times per week	1-4 times per week	1-4 times per week	>=3 meal per day	not daily	daily	no	no snack	not daily	daily	not daily	not daily
three or less times monthly	three or less times monthly	three or less times monthly	>=3 meal per day	not daily	not daily	no	no snack	daily	not daily	not daily	daily
three or less times monthly	three or less times monthly	three or less times monthly	>=3 meal per day	not daily	daily	no	no snack	daily	not daily	never	daily
>=5 times per week	>=5 times per week	three or less times monthly	<3 meal per day	not daily	daily	no	no snack	daily	not daily	not daily	daily
>=5 times per week	>=5 times per week	>=5 times per week	<3 meal per day	not daily	daily	no	no snack	not daily	not daily	not daily	daily
>=5 times per week	>=5 times per week	>=5 times per week	<3 meal per day	daily	not daily	no	no snack	daily	not daily	daily	not daily
three or less times monthly	three or less times monthly	1-4 times per week	>=3 meal per day	not daily	daily	no	no snack	daily	not daily	not daily	not daily
three or less times monthly	1-4 times per week	three or less times monthly	>=3 meal per day	not daily	daily	no	no snack	not daily	not daily	never	daily
>=5 times per week	1-4 times per week	three or less times monthly	>=3 meal per day	daily	daily	no	no snack	daily	not daily	not daily	daily
three or less times monthly	three or less times monthly	three or less times monthly	>=3 meal per day	not daily	daily	no	no snack	daily	not daily	never	daily
three or less times monthly	three or less times monthly	three or less times monthly	>=3 meal per day	not daily	daily	no	no snack	daily	daily	daily	not daily
three or less times monthly	three or less times monthly	1-4 times per week	>=3 meal per day	daily	daily	no	no snack	daily	not daily	never	daily
three or less times monthly	three or less times monthly	three or less times monthly	<3 meal per day	daily	daily	no	no snack	daily	not daily	not daily	daily
three or less times monthly	three or less times monthly	three or less times monthly	>=3 meal per day	not daily	daily	no	no snack	daily	not daily	not daily	daily

>=5 times per week	three or less times monthly	three or less times monthly	>=3 meal per day	daily	daily	no	no snack	daily	not daily	not daily	daily
>=5 times per week	>=5 times per week	1-4 times per week	>=3 meal per day	daily	daily	no	no snack	daily	not daily	daily	not daily
1-4 times per week	three or less times monthly	1-4 times per week	>=3 meal per day	not daily	daily	no	no snack	daily	not daily	not daily	not daily
three or less times monthly	three or less times monthly	1-4 times per week	>=3 meal per day	not daily	not daily	no	no snack	daily	not daily	not daily	daily
1-4 times per week	three or less times monthly	1-4 times per week	>=3 meal per day	daily	daily	no	no snack	daily	not daily	not daily	daily
three or less times monthly	three or less times monthly	three or less times monthly	>=3 meal per day	not daily	daily	no	no snack	daily	not daily	not daily	daily
three or less times monthly	>=5 times per week	three or less times monthly	>=3 meal per day	not daily	daily	no	no snack	not daily	not daily	not daily	daily
three or less times monthly	1-4 times per week	three or less times monthly	<3 meal per day	not daily	daily	no	no snack	daily	not daily	daily	daily
three or less times monthly	three or less times monthly	three or less times monthly	>=3 meal per day	daily	daily	no	no snack	daily	not daily	not daily	not daily
three or less times monthly	three or less times monthly	1-4 times per week	>=3 meal per day	not daily	daily	no	no snack	daily	not daily	not daily	daily
three or less times monthly	three or less times monthly	three or less times monthly	>=3 meal per day	not daily	daily	no	no snack	daily	not daily	not daily	not daily
three or less times monthly	three or less times monthly	three or less times monthly	>=3 meal per day	not daily	daily	no	no snack	daily	not daily	not daily	daily
three or less times monthly	three or less times monthly	three or less times monthly	>=3 meal per day	not daily	daily	no	no snack	daily	not daily	not daily	daily
>=5 times per week	1-4 times per week	three or less times monthly	>=3 meal per day	not daily	daily	no	no snack	daily	not daily	not daily	not daily
>=5 times per week	1-4 times per week	three or less times monthly	<3 meal per day	daily	daily	no	no snack	not daily	not daily	not daily	daily

three or less times monthly	>=5 times per week	>=5 times per week	>=3 meal per day	daily	daily	no	no snack	daily	not daily	never	daily
three or less times monthly	three or less times monthly	three or less times monthly	>=3 meal per day	not daily	daily	no	no snack	not daily	not daily	never	daily
>=5 times per week	1-4 times per week	three or less times monthly	>=3 meal per day	not daily	daily	no	no snack	daily	not daily	not daily	not daily
three or less times monthly	three or less times monthly	three or less times monthly	>=3 meal per day	daily	daily	no	no snack	not daily	not daily	daily	daily
>=5 times per week	three or less times monthly	three or less times monthly	<3 meal per day	not daily	not daily	no	no snack	not daily	not daily	not daily	not daily
1-4 times per week	three or less times monthly	three or less times monthly	>=3 meal per day	not daily	daily	no	no snack	daily	not daily	never	daily
three or less times monthly	three or less times monthly	three or less times monthly	<3 meal per day	daily	daily	no	no snack	daily	not daily	not daily	not daily
1-4 times per week	three or less times monthly	three or less times monthly	>=3 meal per day	not daily	daily	no	no snack	not daily	not daily	not daily	daily
three or less times monthly	1-4 times per week	1-4 times per week	>=3 meal per day	daily	not daily	yes	<=2 per day	not daily	not daily	not daily	daily
three or less times monthly	three or less times monthly	1-4 times per week	<3 meal per day	not daily	daily	yes	<=2 per day	daily	not daily	not daily	daily
>=5 times per week	three or less times monthly	three or less times monthly	<3 meal per day	daily	not daily	yes	<=2 per day	not daily	daily	not daily	daily
three or less times monthly	three or less times monthly	three or less times monthly	>=3 meal per day	not daily	daily	yes	<=2 per day	daily	not daily	not daily	daily
>=5 times per week	1-4 times per week	three or less times monthly	>=3 meal per day	daily	daily	yes	<=2 per day	daily	not daily	not daily	daily
>=5 times per week	1-4 times per week	1-4 times per week	>=3 meal per day	not daily	daily	yes	<=2 per day	daily	not daily	not daily	daily

1-4 times per week	1-4 times per week	three or less times monthly	>=3 meal per day	daily	daily	yes	<=2 per day	daily	not daily	not daily	not daily
>=5 times per week	1-4 times per week	three or less times monthly	>=3 meal per day	not daily	daily	yes	<=2 per day	daily	daily	not daily	daily
>=5 times per week	1-4 times per week	1-4 times per week	>=3 meal per day	daily	daily	yes	<=2 per day	daily	not daily	not daily	not daily
three or less times monthly	three or less times monthly	three or less times monthly	>=3 meal per day	not daily	daily	yes	<=2 per day	daily	not daily	not daily	daily
three or less times monthly	three or less times monthly	three or less times monthly	>=3 meal per day	not daily	daily	yes	<=2 per day	daily	not daily	not daily	daily
>=5 times per week	three or less times monthly	three or less times monthly	>=3 meal per day	not daily	daily	yes	<=2 per day	not daily	daily	daily	daily
three or less times monthly	1-4 times per week	three or less times monthly	>=3 meal per day	not daily	daily	yes	<=2 per day	daily	not daily	not daily	not daily
three or less times monthly	three or less times monthly	three or less times monthly	>=3 meal per day	not daily	daily	yes	<=2 per day	not daily	not daily	daily	daily
three or less times monthly	1-4 times per week	1-4 times per week	>=3 meal per day	not daily	daily	yes	<=2 per day	daily	not daily	not daily	daily
>=5 times per week	1-4 times per week	three or less times monthly	>=3 meal per day	not daily	daily	yes	<=2 per day	daily	not daily	not daily	daily
>=5 times per week	three or less times monthly	three or less times monthly	>=3 meal per day	not daily	daily	yes	<=2 per day	daily	daily	daily	not daily
1-4 times per week	three or less times monthly	three or less times monthly	>=3 meal per day	not daily	daily	yes	<=2 per day	daily	not daily	not daily	daily
>=5 times per week	>=5 times per week	1-4 times per week	>=3 meal per day	daily	daily	yes	<=2 per day	daily	not daily	not daily	daily
1-4 times per week	three or less times monthly	1-4 times per week	>=3 meal per day	daily	not daily	yes	<=2 per day	not daily	not daily	not daily	not daily
1-4 times per week	>=5 times per week	1-4 times per week	>=3 meal per day	not daily	daily	yes	<=2 per day	daily	not daily	not daily	daily

three or less times monthly	1-4 times per week	three or less times monthly	>=3 meal per day	not daily	daily	yes	<=2 per day	daily	not daily	not daily	daily
>=5 times per week	1-4 times per week	>=5 times per week	>=3 meal per day	daily	daily	yes	<=2 per day	daily	not daily	not daily	daily
>=5 times per week	>=5 times per week	three or less times monthly	>=3 meal per day	not daily	daily	yes	<=2 per day	daily	not daily	not daily	daily
>=5 times per week	three or less times monthly	three or less times monthly	>=3 meal per day	daily	daily	yes	<=2 per day	daily	not daily	never	daily
1-4 times per week	three or less times monthly	three or less times monthly	>=3 meal per day	not daily	daily	yes	<=2 per day	daily	not daily	never	daily
1-4 times per week	three or less times monthly	three or less times monthly	>=3 meal per day	not daily	daily	yes	<=2 per day	daily	not daily	not daily	daily
1-4 times per week	three or less times monthly	three or less times monthly	>=3 meal per day	not daily	daily	yes	<=2 per day	daily	not daily	not daily	daily
>=5 times per week	three or less times monthly	three or less times monthly	>=3 meal per day	daily	not daily	yes	<=2 per day	daily	not daily	never	daily
>=5 times per week	1-4 times per week	three or less times monthly	>=3 meal per day	not daily	daily	yes	<=2 per day	daily	not daily	not daily	not daily
1-4 times per week	three or less times monthly	three or less times monthly	>=3 meal per day	not daily	daily	yes	<=2 per day	not daily	daily	not daily	daily
three or less times monthly	three or less times monthly	three or less times monthly	<3 meal per day	not daily	not daily	yes	<=2 per day	not daily	not daily	not daily	daily
1-4 times per week	three or less times monthly	three or less times monthly	>=3 meal per day	not daily	daily	yes	<=2 per day	daily	not daily	not daily	daily
three or less times monthly	three or less times monthly	1-4 times per week	>=3 meal per day	not daily	daily	yes	<=2 per day	daily	not daily	not daily	daily
1-4 times per week	>=5 times per week	three or less times monthly	>=3 meal per day	not daily	daily	yes	<=2 per day	daily	not daily	not daily	daily

>=5 times per week	>=5 times per week	1-4 times per week	>=3 meal per day	not daily	daily	yes	<=2 per day	daily	not daily	not daily	daily
three or less times monthly	three or less times monthly	1-4 times per week	<3 meal per day	not daily	daily	yes	<=2 per day	not daily	not daily	not daily	not daily
1-4 times per week	three or less times monthly	three or less times monthly	>=3 meal per day	not daily	daily	yes	<=2 per day	daily	not daily	not daily	daily
>=5 times per week	1-4 times per week	1-4 times per week	>=3 meal per day	daily	daily	yes	<=2 per day	daily	not daily	not daily	daily
1-4 times per week	1-4 times per week	1-4 times per week	>=3 meal per day	daily	daily	yes	<=2 per day	daily	not daily	not daily	daily
>=5 times per week	1-4 times per week	1-4 times per week	>=3 meal per day	not daily	daily	yes	<=2 per day	daily	not daily	not daily	not daily
>=5 times per week	>=5 times per week	three or less times monthly	>=3 meal per day	daily	daily	yes	<=2 per day	daily	not daily	not daily	daily
three or less times monthly	three or less times monthly	1-4 times per week	>=3 meal per day	not daily	daily	yes	<=2 per day	daily	not daily	not daily	daily
three or less times monthly	three or less times monthly	three or less times monthly	>=3 meal per day	not daily	not daily	yes	<=2 per day	daily	not daily	not daily	daily
>=5 times per week	1-4 times per week	1-4 times per week	>=3 meal per day	daily	daily	yes	<=2 per day	daily	not daily	not daily	not daily
>=5 times per week	1-4 times per week	1-4 times per week	>=3 meal per day	daily	daily	yes	<=2 per day	daily	daily	never	daily
1-4 times per week	three or less times monthly	1-4 times per week	>=3 meal per day	daily	daily	yes	<=2 per day	daily	not daily	not daily	daily
>=5 times per week	>=5 times per week	1-4 times per week	>=3 meal per day	daily	daily	yes	<=2 per day	daily	not daily	not daily	daily
three or less times monthly	three or less times monthly	three or less times monthly	>=3 meal per day	not daily	daily	yes	>=3 per day	daily	not daily	never	daily
three or less times monthly	three or less times monthly	three or less times monthly	>=3 meal per day	daily	daily	yes	>=3 per day	daily	not daily	not daily	not daily

three or less times monthly	three or less times monthly	three or less times monthly	>=3 meal per day	daily	daily	yes	>=3 per day	daily	not daily	daily	daily
three or less times monthly	three or less times monthly	1-4 times per week	>=3 meal per day	daily	daily	yes	>=3 per day	daily	not daily	not daily	daily
1-4 times per week	1-4 times per week	1-4 times per week	>=3 meal per day	not daily	daily	yes	>=3 per day	not daily	not daily	not daily	daily
three or less times monthly	three or less times monthly	1-4 times per week	>=3 meal per day	not daily	daily	yes	>=3 per day	daily	not daily	not daily	daily
three or less times monthly	three or less times monthly	three or less times monthly	>=3 meal per day	not daily	daily	yes	>=3 per day	daily	not daily	not daily	daily
three or less times monthly	three or less times monthly	three or less times monthly	>=3 meal per day	not daily	daily	yes	>=3 per day	daily	daily	not daily	daily
>=5 times per week	>=5 times per week	1-4 times per week	>=3 meal per day	daily	daily	yes	>=3 per day	daily	not daily	not daily	daily
three or less times monthly	three or less times monthly	three or less times monthly	>=3 meal per day	daily	daily	yes	>=3 per day	daily	not daily	never	daily
1-4 times per week	1-4 times per week	three or less times monthly	>=3 meal per day	daily	daily	yes	>=3 per day	daily	not daily	not daily	not daily
1-4 times per week	three or less times monthly	three or less times monthly	>=3 meal per day	not daily	daily	yes	>=3 per day	daily	not daily	not daily	daily
>=5 times per week	1-4 times per week	1-4 times per week	>=3 meal per day	not daily	not daily	yes	>=3 per day	not daily	not daily	not daily	daily
>=5 times per week	>=5 times per week	>=5 times per week	<3 meal per day	not daily	not daily	yes	>=3 per day	daily	daily	daily	not daily
1-4 times per week	1-4 times per week	three or less times monthly	>=3 meal per day	not daily	daily	yes	>=3 per day	daily	not daily	not daily	daily
>=5 times per week	1-4 times per week	three or less times monthly	<3 meal per day	not daily	daily	yes	>=3 per day	daily	not daily	not daily	daily

1-4 times per week	1-4 times per week	1-4 times per week	>=3 meal per day	daily	daily	yes	>=3 per day	daily	daily	daily	daily
1-4 times per week	three or less times monthly	1-4 times per week	<3 meal per day	daily	daily	yes	>=3 per day	daily	not daily	never	daily
three or less times monthly	three or less times monthly	three or less times monthly	>=3 meal per day	not daily	daily	yes	>=3 per day	not daily	not daily	never	daily
three or less times monthly	three or less times monthly	three or less times monthly	<3 meal per day	daily	daily	yes	>=3 per day	daily	daily	not daily	daily
>=5 times per week	>=5 times per week	1-4 times per week	>=3 meal per day	daily	daily	yes	>=3 per day	not daily	not daily	not daily	daily
three or less times monthly	three or less times monthly	three or less times monthly	>=3 meal per day	not daily	daily	no	no snack	daily	not daily	never	daily
>=5 times per week	>=5 times per week	three or less times monthly	>=3 meal per day	not daily	daily	no	no snack	daily	not daily	never	daily
three or less times monthly	three or less times monthly	three or less times monthly	>=3 meal per day	not daily	daily	no	no snack	daily	not daily	never	daily
three or less times monthly	three or less times monthly	three or less times monthly	>=3 meal per day	daily	not daily	no	no snack	daily	not daily	never	daily
three or less times monthly	three or less times monthly	three or less times monthly	>=3 meal per day	not daily	not daily	no	no snack	not daily	not daily	not daily	daily
three or less times monthly	three or less times monthly	three or less times monthly	>=3 meal per day	not daily	daily	no	no snack	daily	not daily	never	daily
>=5 times per week	three or less times monthly	three or less times monthly	>=3 meal per day	not daily	daily	no	no snack	daily	not daily	not daily	daily
>=5 times per week	three or less times monthly	three or less times monthly	>=3 meal per day	not daily	daily	no	no snack	daily	not daily	not daily	daily
three or less times monthly	three or less times monthly	three or less times monthly	>=3 meal per day	daily	daily	no	no snack	daily	not daily	not daily	daily

1-4 times per week	1-4 times per week	1-4 times per week	>=3 meal per day	daily	daily	no	no snack	not daily	not daily	not daily	not daily
1-4 times per week	three or less times monthly	three or less times monthly	>=3 meal per day	not daily	daily	no	no snack	daily	not daily	never	daily
three or less times monthly	three or less times monthly	1-4 times per week	>=3 meal per day	not daily	daily	no	no snack	daily	not daily	never	daily
>=5 times per week	three or less times monthly	three or less times monthly	>=3 meal per day	not daily	not daily	no	no snack	daily	not daily	never	daily
>=5 times per week	three or less times monthly	three or less times monthly	>=3 meal per day	not daily	daily	no	no snack	daily	daily	not daily	daily
three or less times monthly	three or less times monthly	three or less times monthly	>=3 meal per day	not daily	daily	no	no snack	daily	not daily	not daily	daily
three or less times monthly	three or less times monthly	three or less times monthly	>=3 meal per day	not daily	daily	no	no snack	daily	not daily	not daily	daily
three or less times monthly	three or less times monthly	three or less times monthly	>=3 meal per day	not daily	daily	no	no snack	daily	not daily	never	daily
1-4 times per week	three or less times monthly	three or less times monthly	>=3 meal per day	not daily	daily	no	no snack	daily	not daily	never	daily
three or less times monthly	1-4 times per week	three or less times monthly	>=3 meal per day	not daily	daily	no	no snack	daily	not daily	not daily	daily
three or less times monthly	three or less times monthly	three or less times monthly	<3 meal per day	not daily	daily	no	no snack	not daily	not daily	not daily	not daily
1-4 times per week	three or less times monthly	1-4 times per week	>=3 meal per day	not daily	daily	no	no snack	daily	not daily	not daily	not daily
three or less times monthly	1-4 times per week	1-4 times per week	>=3 meal per day	not daily	not daily	no	no snack	not daily	not daily	never	daily
three or less times monthly	three or less times monthly	three or less times monthly	>=3 meal per day	not daily	daily	no	no snack	daily	not daily	never	daily

1-4 times per week	1-4 times per week	three or less times monthly	>=3 meal per day	not daily	daily	no	no snack	not daily	daily	not daily	daily
three or less times monthly	three or less times monthly	three or less times monthly	>=3 meal per day	daily	daily	no	no snack	daily	not daily	not daily	not daily
three or less times monthly	three or less times monthly	three or less times monthly	>=3 meal per day	not daily	daily	no	no snack	not daily	not daily	not daily	daily
1-4 times per week	three or less times monthly	three or less times monthly	<3 meal per day	not daily	daily	no	no snack	daily	not daily	never	daily
three or less times monthly	three or less times monthly	three or less times monthly	>=3 meal per day	not daily	daily	no	no snack	daily	not daily	not daily	daily
three or less times monthly	three or less times monthly	three or less times monthly	>=3 meal per day	not daily	daily	no	no snack	daily	not daily	not daily	daily
three or less times monthly	three or less times monthly	three or less times monthly	>=3 meal per day	daily	daily	no	no snack	daily	not daily	daily	not daily
1-4 times per week	three or less times monthly	three or less times monthly	>=3 meal per day	not daily	daily	no	no snack	daily	not daily	not daily	daily
>=5 times per week	1-4 times per week	three or less times monthly	>=3 meal per day	not daily	not daily	no	no snack	daily	daily	not daily	not daily
three or less times monthly	three or less times monthly	three or less times monthly	>=3 meal per day	not daily	daily	no	no snack	not daily	not daily	not daily	daily
1-4 times per week	>=5 times per week	three or less times monthly	>=3 meal per day	not daily	daily	no	no snack	daily	daily	never	daily
1-4 times per week	three or less times monthly	three or less times monthly	>=3 meal per day	not daily	not daily	no	no snack	not daily	not daily	not daily	not daily
>=5 times per week	three or less times monthly	three or less times monthly	>=3 meal per day	daily	daily	no	no snack	daily	not daily	daily	daily
1-4 times per week	three or less times monthly	three or less times monthly	>=3 meal per day	not daily	daily	no	no snack	not daily	not daily	not daily	not daily

1-4 times per week	three or less times monthly	three or less times monthly	>=3 meal per day	not daily	daily	no	no snack	daily	not daily	not daily	daily
>=5 times per week	three or less times monthly	three or less times monthly	>=3 meal per day	not daily	daily	no	no snack	daily	not daily	never	daily
three or less times monthly	three or less times monthly	three or less times monthly	>=3 meal per day	daily	daily	no	no snack	daily	not daily	never	daily
three or less times monthly	1-4 times per week	1-4 times per week	>=3 meal per day	not daily	daily	no	no snack	daily	not daily	never	daily
three or less times monthly	three or less times monthly	three or less times monthly	>=3 meal per day	not daily	daily	no	no snack	daily	not daily	not daily	daily
three or less times monthly	three or less times monthly	three or less times monthly	>=3 meal per day	not daily	daily	no	no snack	daily	not daily	not daily	daily
>=5 times per week	three or less times monthly	three or less times monthly	>=3 meal per day	not daily	not daily	no	no snack	daily	daily	not daily	not daily
three or less times monthly	1-4 times per week	1-4 times per week	>=3 meal per day	not daily	daily	no	no snack	daily	not daily	not daily	daily
1-4 times per week	1-4 times per week	1-4 times per week	>=3 meal per day	daily	daily	no	no snack	daily	daily	never	daily
1-4 times per week	1-4 times per week	1-4 times per week	>=3 meal per day	daily	daily	no	no snack	daily	not daily	not daily	daily
1-4 times per week	three or less times monthly	three or less times monthly	>=3 meal per day	not daily	daily	no	no snack	daily	not daily	not daily	daily
three or less times monthly	three or less times monthly	three or less times monthly	>=3 meal per day	not daily	daily	no	no snack	daily	not daily	not daily	daily
three or less times monthly	three or less times monthly	three or less times monthly	>=3 meal per day	not daily	daily	no	no snack	daily	not daily	not daily	daily
three or less times monthly	three or less times monthly	1-4 times per week	<3 meal per day	daily	daily	no	no snack	daily	not daily	never	daily
three or less times monthly	three or less times monthly	three or less times monthly	>=3 meal per day	daily	daily	no	no snack	daily	daily	not daily	daily

1-4 times per week	three or less times monthly	three or less times monthly	<3 meal per day	not daily	daily	no	no snack	daily	not daily	daily	daily
three or less times monthly	1-4 times per week	three or less times monthly	<3 meal per day	daily	daily	no	no snack	daily	daily	never	daily
1-4 times per week	three or less times monthly	three or less times monthly	>=3 meal per day	daily	daily	no	no snack	daily	not daily	not daily	daily
three or less times monthly	1-4 times per week	>=5 times per week	>=3 meal per day	not daily	daily	no	no snack	daily	not daily	not daily	daily
three or less times monthly	three or less times monthly	three or less times monthly	>=3 meal per day	not daily	daily	no	no snack	daily	not daily	not daily	daily
1-4 times per week	three or less times monthly	three or less times monthly	>=3 meal per day	not daily	daily	no	no snack	not daily	not daily	never	daily
>=5 times per week	three or less times monthly	three or less times monthly	>=3 meal per day	daily	daily	no	no snack	daily	not daily	not daily	daily
three or less times monthly	three or less times monthly	three or less times monthly	>=3 meal per day	not daily	daily	no	no snack	daily	not daily	not daily	daily
three or less times monthly	1-4 times per week	three or less times monthly	>=3 meal per day	not daily	daily	no	no snack	daily	not daily	never	daily
three or less times monthly	three or less times monthly	three or less times monthly	<3 meal per day	not daily	daily	no	no snack	daily	not daily	not daily	not daily
three or less times monthly	three or less times monthly	three or less times monthly	>=3 meal per day	not daily	daily	no	no snack	daily	daily	daily	not daily
>=5 times per week	three or less times monthly	1-4 times per week	>=3 meal per day	not daily	daily	no	no snack	daily	not daily	never	daily
1-4 times per week	1-4 times per week	>=5 times per week	>=3 meal per day	not daily	daily	no	no snack	not daily	not daily	never	daily
1-4 times per week	1-4 times per week	three or less times monthly	>=3 meal per day	not daily	daily	no	no snack	daily	not daily	not daily	daily
three or less times monthly	three or less times monthly	three or less times monthly	>=3 meal per day	not daily	daily	no	no snack	daily	not daily	not daily	daily

1-4 times per week	three or less times monthly	three or less times monthly	>=3 meal per day	not daily	daily	no	no snack	daily	not daily	not daily	not daily
>=5 times per week	1-4 times per week	three or less times monthly	>=3 meal per day	not daily	not daily	no	no snack	daily	not daily	never	daily
three or less times monthly	three or less times monthly	three or less times monthly	>=3 meal per day	not daily	daily	no	no snack	daily	not daily	not daily	daily
1-4 times per week	three or less times monthly	three or less times monthly	>=3 meal per day	daily	daily	no	no snack	daily	daily	not daily	not daily
>=5 times per week	1-4 times per week	1-4 times per week	>=3 meal per day	daily	daily	no	no snack	daily	daily	not daily	not daily
three or less times monthly	1-4 times per week	1-4 times per week	>=3 meal per day	not daily	not daily	no	no snack	daily	not daily	not daily	daily
1-4 times per week	three or less times monthly	three or less times monthly	>=3 meal per day	daily	daily	no	no snack	daily	not daily	not daily	daily
1-4 times per week	three or less times monthly	>=5 times per week	>=3 meal per day	daily	daily	no	no snack	not daily	not daily	never	daily
>=5 times per week	three or less times monthly	three or less times monthly	>=3 meal per day	daily	daily	no	no snack	daily	not daily	never	daily
>=5 times per week	three or less times monthly	three or less times monthly	<3 meal per day	not daily	daily	no	no snack	not daily	not daily	not daily	daily
three or less times monthly	three or less times monthly	three or less times monthly	<3 meal per day	not daily	not daily	no	no snack	not daily	not daily	never	daily
1-4 times per week	1-4 times per week	three or less times monthly	>=3 meal per day	not daily	daily	no	no snack	daily	not daily	not daily	daily
1-4 times per week	three or less times monthly	three or less times monthly	>=3 meal per day	not daily	daily	no	no snack	not daily	daily	not daily	daily
three or less times monthly	>=5 times per week	three or less times monthly	>=3 meal per day	not daily	not daily	no	no snack	daily	not daily	never	not daily

three or less times monthly	three or less times monthly	three or less times monthly	>=3 meal per day	daily	daily	no	no snack	daily	not daily	never	daily
three or less times monthly	three or less times monthly	three or less times monthly	<3 meal per day	not daily	daily	no	no snack	daily	not daily	not daily	daily
three or less times monthly	three or less times monthly	>=5 times per week	>=3 meal per day	daily	daily	no	no snack	daily	not daily	not daily	daily
three or less times monthly	three or less times monthly	1-4 times per week	>=3 meal per day	not daily	daily	no	no snack	not daily	not daily	not daily	daily
three or less times monthly	three or less times monthly	1-4 times per week	<3 meal per day	not daily	not daily	no	no snack	daily	not daily	daily	not daily
1-4 times per week	1-4 times per week	1-4 times per week	>=3 meal per day	not daily	not daily	no	no snack	not daily	not daily	not daily	not daily
>=5 times per week	three or less times monthly	three or less times monthly	>=3 meal per day	daily	daily	no	no snack	daily	not daily	not daily	not daily
1-4 times per week	three or less times monthly	three or less times monthly	>=3 meal per day	not daily	daily	no	no snack	daily	not daily	not daily	daily
three or less times monthly	three or less times monthly	three or less times monthly	>=3 meal per day	not daily	daily	no	no snack	daily	not daily	not daily	daily
1-4 times per week	three or less times monthly	three or less times monthly	>=3 meal per day	daily	daily	no	no snack	daily	not daily	not daily	daily
three or less times monthly	three or less times monthly	three or less times monthly	>=3 meal per day	not daily	daily	no	no snack	daily	not daily	not daily	daily
1-4 times per week	three or less times monthly	three or less times monthly	>=3 meal per day	not daily	daily	no	no snack	daily	not daily	not daily	daily
three or less times monthly	1-4 times per week	three or less times monthly	>=3 meal per day	not daily	daily	no	no snack	daily	not daily	not daily	daily
1-4 times per week	three or less times monthly	three or less times monthly	>=3 meal per day	daily	daily	no	no snack	daily	not daily	not daily	daily

three or less times monthly	three or less times monthly	three or less times monthly	>=3 meal per day	not daily	daily	no	no snack	daily	not daily	daily	daily
>=5 times per week	>=5 times per week	three or less times monthly	>=3 meal per day	not daily	daily	no	no snack	daily	not daily	not daily	daily
>=5 times per week	>=5 times per week	three or less times monthly	>=3 meal per day	not daily	daily	no	no snack	daily	not daily	not daily	daily
1-4 times per week	1-4 times per week	>=5 times per week	<3 meal per day	daily	not daily	no	no snack	daily	not daily	never	daily
three or less times monthly	three or less times monthly	three or less times monthly	>=3 meal per day	not daily	daily	no	no snack	daily	not daily	never	daily
three or less times monthly	three or less times monthly	three or less times monthly	>=3 meal per day	daily	not daily	no	no snack	daily	not daily	never	daily
1-4 times per week	>=5 times per week	three or less times monthly	>=3 meal per day	daily	not daily	no	no snack	not daily	not daily	not daily	daily
1-4 times per week	three or less times monthly	three or less times monthly	>=3 meal per day	not daily	daily	no	no snack	daily	not daily	never	daily
1-4 times per week	1-4 times per week	three or less times monthly	>=3 meal per day	not daily	daily	no	no snack	daily	not daily	not daily	daily
three or less times monthly	three or less times monthly	three or less times monthly	>=3 meal per day	not daily	daily	no	no snack	daily	not daily	not daily	daily
three or less times monthly	three or less times monthly	three or less times monthly	>=3 meal per day	not daily	daily	no	no snack	daily	not daily	not daily	daily
three or less times monthly	three or less times monthly	three or less times monthly	>=3 meal per day	not daily	daily	no	no snack	daily	not daily	not daily	daily
three or less times monthly	three or less times monthly	three or less times monthly	>=3 meal per day	not daily	daily	no	no snack	daily	not daily	not daily	daily
>=5 times per week	three or less times monthly	>=5 times per week	>=3 meal per day	not daily	daily	no	no snack	not daily	daily	daily	not daily

1-4 times per week	three or less times monthly	1-4 times per week	>=3 meal per day	not daily	daily	no	no snack	daily	not daily	not daily	daily
three or less times monthly	three or less times monthly	three or less times monthly	>=3 meal per day	not daily	daily	no	no snack	daily	not daily	not daily	daily
>=5 times per week	three or less times monthly	three or less times monthly	>=3 meal per day	not daily	daily	no	no snack	daily	not daily	not daily	not daily
1-4 times per week	three or less times monthly	three or less times monthly	>=3 meal per day	not daily	daily	no	no snack	daily	not daily	not daily	daily
>=5 times per week	1-4 times per week	three or less times monthly	>=3 meal per day	not daily	daily	no	no snack	daily	not daily	not daily	daily
>=5 times per week	three or less times monthly	three or less times monthly	>=3 meal per day	not daily	daily	no	no snack	daily	not daily	not daily	daily
three or less times monthly	three or less times monthly	three or less times monthly	>=3 meal per day	daily	daily	no	no snack	daily	not daily	not daily	daily
three or less times monthly	three or less times monthly	1-4 times per week	>=3 meal per day	daily	daily	no	no snack	daily	not daily	not daily	daily
three or less times monthly	three or less times monthly	three or less times monthly	>=3 meal per day	not daily	daily	no	no snack	daily	not daily	not daily	daily
>=5 times per week	>=5 times per week	three or less times monthly	>=3 meal per day	not daily	daily	no	no snack	daily	not daily	not daily	not daily
1-4 times per week	1-4 times per week	>=5 times per week	>=3 meal per day	daily	daily	no	no snack	daily	not daily	never	daily
1-4 times per week	three or less times monthly	three or less times monthly	>=3 meal per day	not daily	daily	no	no snack	daily	not daily	not daily	daily
>=5 times per week	three or less times monthly	three or less times monthly	>=3 meal per day	not daily	daily	no	no snack	daily	not daily	not daily	daily
three or less times monthly	>=5 times per week	three or less times monthly	>=3 meal per day	daily	daily	no	no snack	daily	not daily	not daily	daily

1-4 times per week	three or less times monthly	three or less times monthly	>=3 meal per day	daily	daily	no	no snack	daily	not daily	not daily	daily
three or less times monthly	three or less times monthly	three or less times monthly	>=3 meal per day	not daily	daily	no	no snack	daily	not daily	not daily	daily
three or less times monthly	three or less times monthly	1-4 times per week	>=3 meal per day	not daily	daily	no	no snack	daily	not daily	never	daily
three or less times monthly	1-4 times per week	three or less times monthly	>=3 meal per day	daily	daily	no	no snack	daily	not daily	not daily	daily
>=5 times per week	>=5 times per week	>=5 times per week	>=3 meal per day	daily	daily	no	no snack	daily	not daily	not daily	not daily
1-4 times per week	three or less times monthly	three or less times monthly	>=3 meal per day	not daily	not daily	no	no snack	daily	not daily	not daily	daily
three or less times monthly	three or less times monthly	three or less times monthly	>=3 meal per day	daily	daily	no	no snack	daily	not daily	not daily	daily
>=5 times per week	1-4 times per week	>=5 times per week	>=3 meal per day	daily	daily	no	no snack	daily	not daily	never	daily
1-4 times per week	1-4 times per week	1-4 times per week	>=3 meal per day	not daily	daily	no	no snack	daily	not daily	not daily	daily
>=5 times per week	1-4 times per week	1-4 times per week	>=3 meal per day	not daily	daily	no	no snack	daily	not daily	not daily	daily
>=5 times per week	1-4 times per week	1-4 times per week	>=3 meal per day	not daily	daily	no	no snack	daily	not daily	not daily	daily
three or less times monthly	three or less times monthly	three or less times monthly	>=3 meal per day	daily	daily	no	no snack	daily	not daily	not daily	not daily
three or less times monthly	three or less times monthly	1-4 times per week	>=3 meal per day	not daily	not daily	no	no snack	daily	not daily	not daily	daily
three or less times monthly	three or less times monthly	three or less times monthly	>=3 meal per day	not daily	daily	no	no snack	daily	not daily	not daily	daily

1-4 times per week	three or less times monthly	three or less times monthly	>=3 meal per day	not daily	daily	no	no snack	not daily	not daily	not daily	daily
1-4 times per week	three or less times monthly	three or less times monthly	>=3 meal per day	not daily	daily	no	no snack	daily	not daily	not daily	daily
1-4 times per week	>=5 times per week	three or less times monthly	>=3 meal per day	not daily	daily	no	no snack	daily	not daily	not daily	daily
three or less times monthly	three or less times monthly	three or less times monthly	>=3 meal per day	not daily	daily	no	no snack	daily	not daily	not daily	daily
three or less times monthly	three or less times monthly	1-4 times per week	>=3 meal per day	not daily	daily	no	no snack	daily	not daily	not daily	daily
1-4 times per week	three or less times monthly	three or less times monthly	<3 meal per day	not daily	daily	no	no snack	not daily	not daily	not daily	not daily
1-4 times per week	1-4 times per week	1-4 times per week	>=3 meal per day	not daily	daily	no	no snack	daily	not daily	not daily	daily
1-4 times per week	three or less times monthly	three or less times monthly	>=3 meal per day	not daily	daily	no	no snack	daily	not daily	not daily	daily
>=5 times per week	1-4 times per week	>=5 times per week	>=3 meal per day	not daily	not daily	no	no snack	daily	not daily	not daily	daily
three or less times monthly	three or less times monthly	three or less times monthly	>=3 meal per day	not daily	daily	no	no snack	daily	not daily	not daily	daily
three or less times monthly	three or less times monthly	three or less times monthly	>=3 meal per day	not daily	daily	no	no snack	daily	not daily	not daily	daily
three or less times monthly	three or less times monthly	1-4 times per week	<3 meal per day	not daily	not daily	no	no snack	daily	not daily	never	daily
1-4 times per week	three or less times monthly	three or less times monthly	>=3 meal per day	not daily	daily	no	no snack	daily	not daily	not daily	daily
three or less times monthly	three or less times monthly	three or less times monthly	>=3 meal per day	not daily	daily	no	no snack	daily	not daily	not daily	not daily

>=5 times per week	>=5 times per week	three or less times monthly	>=3 meal per day	not daily	daily	no	no snack	daily	not daily	not daily	daily
three or less times monthly	three or less times monthly	three or less times monthly	>=3 meal per day	not daily	not daily	no	no snack	daily	daily	never	daily
three or less times monthly	three or less times monthly	three or less times monthly	>=3 meal per day	daily	daily	no	no snack	daily	not daily	not daily	daily
three or less times monthly	three or less times monthly	three or less times monthly	>=3 meal per day	daily	daily	no	no snack	daily	not daily	daily	daily
three or less times monthly	three or less times monthly	three or less times monthly	>=3 meal per day	not daily	daily	yes	<=2 per day	daily	daily	daily	daily
three or less times monthly	three or less times monthly	three or less times monthly	>=3 meal per day	not daily	daily	yes	<=2 per day	daily	not daily	not daily	daily
>=5 times per week	>=5 times per week	>=5 times per week	>=3 meal per day	not daily	not daily	yes	<=2 per day	not daily	not daily	never	not daily
1-4 times per week	three or less times monthly	three or less times monthly	>=3 meal per day	daily	daily	yes	<=2 per day	daily	not daily	not daily	daily
three or less times monthly	three or less times monthly	three or less times monthly	>=3 meal per day	not daily	daily	yes	<=2 per day	daily	not daily	never	daily
>=5 times per week	1-4 times per week	three or less times monthly	>=3 meal per day	not daily	daily	yes	<=2 per day	daily	not daily	not daily	daily
three or less times monthly	three or less times monthly	three or less times monthly	>=3 meal per day	daily	daily	yes	<=2 per day	daily	not daily	never	daily
>=5 times per week	>=5 times per week	1-4 times per week	>=3 meal per day	not daily	not daily	yes	<=2 per day	daily	not daily	not daily	daily
1-4 times per week	three or less times monthly	three or less times monthly	>=3 meal per day	daily	daily	yes	<=2 per day	daily	not daily	not daily	daily
three or less times monthly	>=5 times per week	1-4 times per week	>=3 meal per day	not daily	daily	yes	<=2 per day	daily	daily	daily	daily

>=5 times per week	three or less times monthly	three or less times monthly	>=3 meal per day	not daily	daily	yes	<=2 per day	daily	not daily	not daily	daily
>=5 times per week	1-4 times per week	>=5 times per week	>=3 meal per day	not daily	daily	yes	<=2 per day	daily	not daily	not daily	not daily
three or less times monthly	1-4 times per week	1-4 times per week	>=3 meal per day	not daily	daily	yes	<=2 per day	daily	not daily	never	daily
three or less times monthly	three or less times monthly	>=5 times per week	>=3 meal per day	not daily	daily	yes	<=2 per day	daily	not daily	never	daily
>=5 times per week	>=5 times per week	1-4 times per week	>=3 meal per day	daily	daily	yes	<=2 per day	daily	not daily	not daily	daily
1-4 times per week	>=5 times per week	1-4 times per week	>=3 meal per day	not daily	daily	yes	<=2 per day	daily	not daily	not daily	daily
1-4 times per week	three or less times monthly	1-4 times per week	>=3 meal per day	not daily	daily	yes	<=2 per day	daily	not daily	never	daily
>=5 times per week	three or less times monthly	three or less times monthly	<3 meal per day	not daily	daily	yes	<=2 per day	not daily	not daily	not daily	not daily
three or less times monthly	three or less times monthly	three or less times monthly	>=3 meal per day	daily	not daily	yes	<=2 per day	daily	not daily	not daily	not daily
>=5 times per week	three or less times monthly	three or less times monthly	>=3 meal per day	not daily	daily	yes	<=2 per day	daily	not daily	not daily	daily
1-4 times per week	1-4 times per week	three or less times monthly	>=3 meal per day	daily	daily	yes	<=2 per day	daily	not daily	never	daily
1-4 times per week	three or less times monthly	three or less times monthly	>=3 meal per day	not daily	daily	yes	<=2 per day	daily	not daily	not daily	daily
>=5 times per week	three or less times monthly	three or less times monthly	>=3 meal per day	daily	daily	yes	<=2 per day	not daily	daily	never	daily
three or less times monthly	three or less times monthly	three or less times monthly	<3 meal per day	not daily	daily	yes	<=2 per day	daily	not daily	not daily	daily

three or less times monthly	three or less times monthly	three or less times monthly	>=3 meal per day	daily	daily	yes	<=2 per day	daily	daily	not daily	daily
three or less times monthly	three or less times monthly	three or less times monthly	>=3 meal per day	not daily	daily	yes	<=2 per day	daily	not daily	not daily	daily
three or less times monthly	three or less times monthly	three or less times monthly	>=3 meal per day	not daily	daily	yes	<=2 per day	daily	not daily	not daily	daily
1-4 times per week	three or less times monthly	three or less times monthly	>=3 meal per day	not daily	daily	yes	<=2 per day	daily	not daily	not daily	daily
three or less times monthly	three or less times monthly	three or less times monthly	>=3 meal per day	not daily	daily	yes	<=2 per day	daily	not daily	not daily	daily
1-4 times per week	1-4 times per week	1-4 times per week	>=3 meal per day	daily	daily	yes	<=2 per day	daily	daily	never	daily
1-4 times per week	1-4 times per week	1-4 times per week	>=3 meal per day	not daily	daily	yes	<=2 per day	daily	not daily	not daily	daily
1-4 times per week	1-4 times per week	1-4 times per week	>=3 meal per day	not daily	daily	yes	<=2 per day	daily	not daily	not daily	daily
1-4 times per week	three or less times monthly	three or less times monthly	>=3 meal per day	not daily	daily	yes	<=2 per day	daily	not daily	not daily	daily
1-4 times per week	three or less times monthly	three or less times monthly	>=3 meal per day	not daily	daily	yes	<=2 per day	daily	not daily	not daily	daily
>=5 times per week	>=5 times per week	>=5 times per week	>=3 meal per day	not daily	daily	yes	<=2 per day	not daily	daily	not daily	daily
three or less times monthly	three or less times monthly	three or less times monthly	>=3 meal per day	not daily	not daily	yes	<=2 per day	not daily	not daily	never	daily
three or less times monthly	1-4 times per week	three or less times monthly	>=3 meal per day	not daily	not daily	yes	<=2 per day	daily	not daily	not daily	daily
three or less times monthly	1-4 times per week	1-4 times per week	>=3 meal per day	daily	daily	yes	<=2 per day	daily	daily	daily	not daily

>=5 times per week	three or less times monthly	three or less times monthly	>=3 meal per day	daily	daily	yes	<=2 per day	daily	not daily	not daily	daily
>=5 times per week	>=5 times per week	>=5 times per week	>=3 meal per day	not daily	not daily	yes	<=2 per day	daily	not daily	not daily	not daily
1-4 times per week	three or less times monthly	three or less times monthly	>=3 meal per day	daily	daily	yes	<=2 per day	daily	daily	never	daily
1-4 times per week	three or less times monthly	1-4 times per week	>=3 meal per day	not daily	daily	yes	<=2 per day	daily	not daily	not daily	daily
three or less times monthly	three or less times monthly	three or less times monthly	>=3 meal per day	daily	daily	yes	<=2 per day	daily	not daily	not daily	daily
1-4 times per week	>=5 times per week	1-4 times per week	>=3 meal per day	daily	daily	yes	<=2 per day	daily	not daily	not daily	daily
1-4 times per week	three or less times monthly	three or less times monthly	>=3 meal per day	not daily	daily	yes	<=2 per day	daily	not daily	not daily	daily
1-4 times per week	three or less times monthly	three or less times monthly	>=3 meal per day	daily	daily	yes	<=2 per day	daily	not daily	never	daily
three or less times monthly	1-4 times per week	1-4 times per week	>=3 meal per day	daily	daily	yes	>=3 per day	daily	not daily	not daily	daily
three or less times monthly	three or less times monthly	>=5 times per week	>=3 meal per day	daily	daily	yes	>=3 per day	daily	not daily	not daily	daily
1-4 times per week	1-4 times per week	three or less times monthly	>=3 meal per day	daily	daily	yes	>=3 per day	daily	not daily	daily	daily
>=5 times per week	three or less times monthly	three or less times monthly	>=3 meal per day	not daily	daily	yes	>=3 per day	daily	not daily	not daily	not daily
three or less times monthly	three or less times monthly	1-4 times per week	>=3 meal per day	not daily	daily	yes	>=3 per day	daily	not daily	not daily	daily
1-4 times per week	1-4 times per week	1-4 times per week	>=3 meal per day	not daily	daily	yes	>=3 per day	daily	not daily	daily	daily
>=5 times per week	1-4 times per week	three or less times monthly	>=3 meal per day	not daily	daily	yes	>=3 per day	daily	not daily	not daily	daily

1-4 times per week	>=5 times per week	1-4 times per week	>=3 meal per day	not daily	daily	yes	>=3 per day	daily	not daily	never	daily
1-4 times per week	1-4 times per week	three or less times monthly	>=3 meal per day	not daily	daily	yes	>=3 per day	daily	daily	not daily	daily
1-4 times per week	three or less times monthly	three or less times monthly	>=3 meal per day	not daily	daily	yes	>=3 per day	daily	not daily	not daily	daily
>=5 times per week	1-4 times per week	1-4 times per week	>=3 meal per day	not daily	daily	yes	>=3 per day	daily	not daily	not daily	daily
1-4 times per week	1-4 times per week	three or less times monthly	>=3 meal per day	not daily	daily	yes	>=3 per day	daily	not daily	never	daily
1-4 times per week	1-4 times per week	1-4 times per week	>=3 meal per day	not daily	daily	yes	>=3 per day	daily	not daily	not daily	daily
1-4 times per week	three or less times monthly	three or less times monthly	>=3 meal per day	not daily	daily	yes	>=3 per day	daily	not daily	never	daily
1-4 times per week	three or less times monthly	1-4 times per week	>=3 meal per day	daily	daily	yes	>=3 per day	daily	not daily	not daily	daily
three or less times monthly	three or less times monthly	three or less times monthly	>=3 meal per day	daily	daily	yes	>=3 per day	daily	not daily	not daily	daily
three or less times monthly	three or less times monthly	three or less times monthly	>=3 meal per day	daily	daily	yes	>=3 per day	daily	not daily	not daily	daily
1-4 times per week	1-4 times per week	three or less times monthly	>=3 meal per day	daily	daily	yes	>=3 per day	daily	not daily	never	daily
three or less times monthly	three or less times monthly	three or less times monthly	>=3 meal per day	not daily	daily	no	no snack	daily	daily	not daily	not daily
three or less times monthly	three or less times monthly	three or less times monthly	>=3 meal per day	not daily	daily	no	no snack	daily	not daily	not daily	daily
1-4 times per week	three or less times monthly	three or less times monthly	>=3 meal per day	not daily	daily	no	no snack	daily	not daily	not daily	not daily

>=5 times per week	three or less times monthly	three or less times monthly	>=3 meal per day	not daily	daily	no	no snack	daily	not daily	not daily	daily
1-4 times per week	three or less times monthly	three or less times monthly	>=3 meal per day	not daily	daily	yes	<=2 per day	daily	not daily	not daily	daily
>=5 times per week	three or less times monthly	three or less times monthly	>=3 meal per day	not daily	daily	yes	<=2 per day	daily	not daily	not daily	daily
1-4 times per week	three or less times monthly	1-4 times per week	>=3 meal per day	daily	daily	yes	<=2 per day	daily	not daily	not daily	not daily
>=5 times per week	three or less times monthly	three or less times monthly	>=3 meal per day	not daily	daily	yes	<=2 per day	daily	not daily	not daily	daily
three or less times monthly	three or less times monthly	three or less times monthly	>=3 meal per day	not daily	daily	no	no snack	daily	daily	not daily	daily
1-4 times per week	three or less times monthly	three or less times monthly	>=3 meal per day	daily	daily	no	no snack	daily	not daily	not daily	daily
three or less times monthly	three or less times monthly	three or less times monthly	>=3 meal per day	not daily	daily	no	no snack	daily	not daily	not daily	daily
1-4 times per week	three or less times monthly	1-4 times per week	>=3 meal per day	daily	daily	yes	<=2 per day	not daily	not daily	not daily	daily
three or less times monthly	three or less times monthly	three or less times monthly	>=3 meal per day	not daily	daily	yes	<=2 per day	daily	not daily	not daily	not daily
1-4 times per week	three or less times monthly	three or less times monthly	>=3 meal per day	not daily	daily	yes	<=2 per day	daily	not daily	not daily	daily

Sheet21650871931166

Sheet31650871931182
